# Archaeological evidence for two culture diverse Neanderthal populations in the North Caucasus and contacts between them

**DOI:** 10.1371/journal.pone.0284093

**Published:** 2023-04-13

**Authors:** Ekaterina V. Doronicheva, Liubov V. Golovanova, Vladimir B. Doronichev, Redzhep N. Kurbanov

**Affiliations:** 1 Autonomous Nonprofit Organization in the Field of Humanitarian and Scientific Research «Laboratory of Prehistory», St. Petersburg, Russia; 2 Institute of Geography RAS, Moscow, Russia; Universita degli Studi di Ferrara, ITALY

## Abstract

Neanderthals were widespread during the Middle Palaeolithic (MP) across Europe and Asia, including the Caucasus Mountains. Occupying the border between eastern Europe and West Asia, the Caucasus is important region regarding the Neanderthal occupation of Eurasia. On current radiometric estimates, the MP is represented in the Caucasus between about 260–210 ka and about 40 ka. Archaeological record indicates that several culture diverse MP hominin populations inhabited the Caucasus, but the region complex population history during this period remains poorly understood. In this paper, we identify for the first time the archaeological evidence indicating contacts between two culture diverse MP Neanderthal populations in the North Caucasus and discuss the nature of these contacts. Basing on the lithic assemblages that we excavated at Mezmaiskaya cave in the north-western Caucasus (Kuban River basin) and Saradj-Chuko grotto in the north-central Caucasus (Terek River basin), dating from MIS 5 to MIS 3, and comparative data from other MP sites in the Caucasus, we identify two large cultural regions that existed during the late MP in the North Caucasus. The distinctive toolkits and stone knapping technologies indicate that the MP assemblages from Mezmaiskaya cave and other sites in the west of North Caucasus represent a Caucasian variant of the Eastern Micoquian industry that was wide spread in central and eastern Europe, while the assemblages from Saradj-Chuko Grotto and other sites in the east of North Caucasus closely resemble the Zagros Mousterian industry that was wide spread in the Armenian Highlands, Lesser Caucasus and Zagros Mountains. The archaeological evidence implies that two culture diverse populations of Neanderthals settled the North Caucasus during the Late Pleistocene from two various source regions: from the Armenian Highlands and Lesser Caucasus along the Caspian Sea coast, and from Russian plain along the Sea of Azov coast.

## Introduction

The Neanderthals were widespread during the Middle Palaeolithic (MP) across Europe and Asia, and also occupied the Caucasus Mountains lying on the border between eastern Europe and south-western Asia. On current radiometric data, MP Neanderthals were present in the Caucasus from ca. 260–210 thousand years ago (kiloannus, ka) to ca. 40 ka [1–6]. Several culture different MP entities, represented by over 270 open-air and cave sites, are identified in the Caucasus [7–9]. Most researchers working in the Caucasus tend to see basic technological and typological distinctions associating the MP sites in the north-western Caucasus with the Eastern Micoquian in central and eastern Europe, and the MP sites in the South and Lesser Caucasus, and the Armenian Highlands with the Levantine Mousterian and Zagros Mousterian in south-western Asia.

However, a complex population history of the region during the MP period remains poorly understood. There is currently little understanding regarding the origins, geographic dispersion and cultural development of various MP entities in the Caucasus, relationship between different Neanderthal groups in this region and with other hominin groups in regions outside the Caucasus, as well as important contributions made by the Caucasian Neanderthals to the dispersal and biocultural evolution of MP hominins in Europe and Asia.

**Fig 1:**
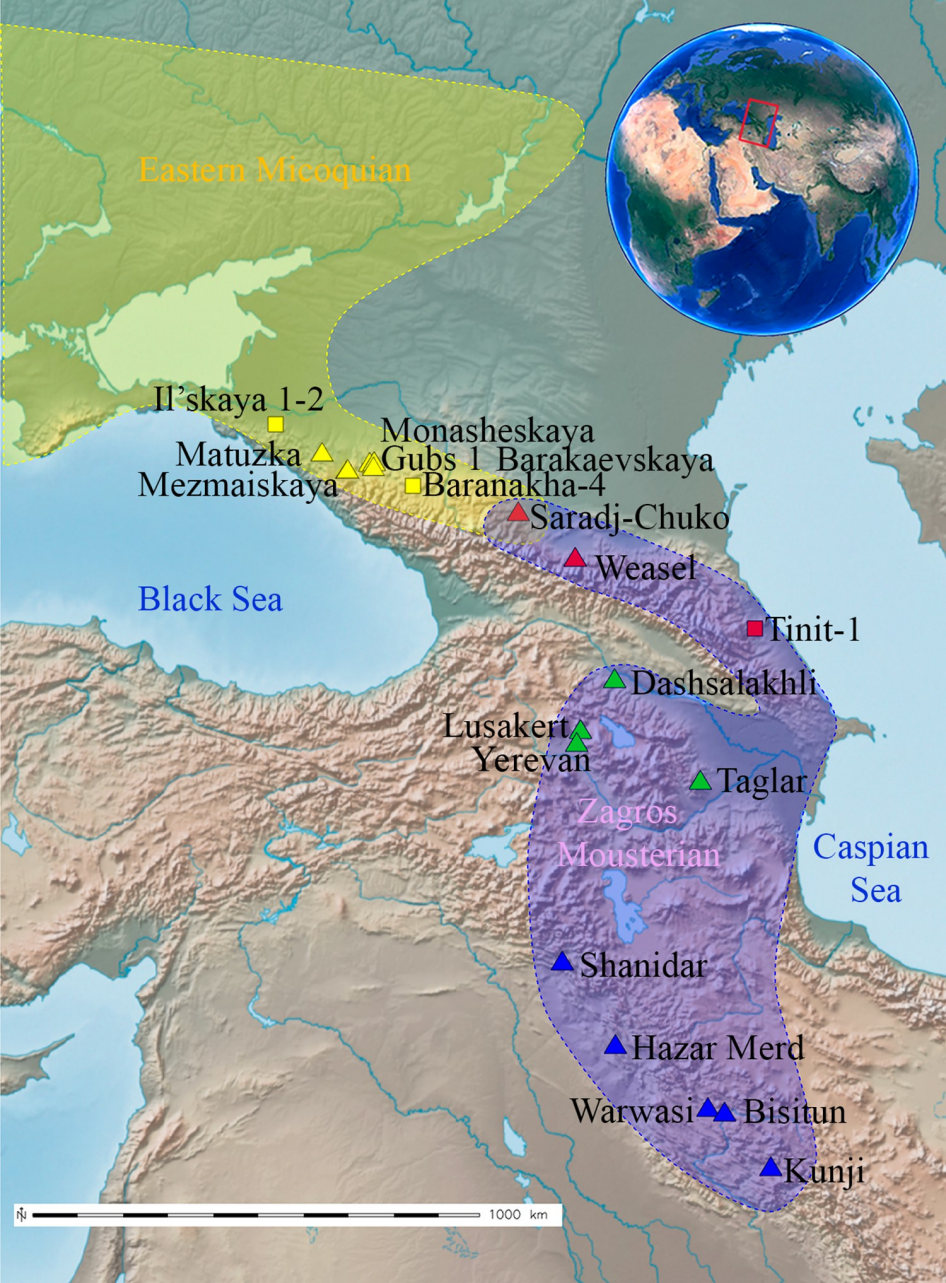
Map showing distribution of the Eastern Micoquian industry in Eastern Europe and the west of North Caucasus and the Zagros Mousterian industry in the Zagros, Caucasus, and Armenian Highlands, including the east of North Caucasus. Squares indicate open-air sites and triangles indicate cave sites. Various colors indicate: yellow–main Eastern Micoquian sites in the west of North Caucasus, red–Zagros Mousterian sites in the east of North Caucasus, green–main Zagros Mousterian sites in the Lesser Caucasus and Armenian Highlands, blue–main Zagros Mousterian sites in the Zagros. Data: Natural Earth (public domain at http://www.naturalearthdata.com). Figure produced using GRASS GIS 7.8 and Inkscape 0.97 software.

In the North Caucasus, previous studies demonstrated that almost all stratified MP sites in the north-western Caucasus (Kuban River basin) represent a regional variant of the Eastern Micoquian industry that was produced by Neanderthals (Fig 1). In the region, there are known 12 stratified sites with about 30 MP layers in total, which are dated from Marine Isotope Stage (MIS) 5d or even MIS 5e to middle MIS 3 [7, 9–12] (Data 1 in S1 Text). The presence of specific types of partly bifacial tools, including small handaxes, leaf-shaped projectile points, various convergent tools and side-scrapers, and asymmetric backed scraper-knives, and non-Levallois and non-laminar recurrent flaking technology, with low indexes of prepared (IF) and faceted (IFs) platforms on flakes are characteristic to the Eastern Micoquian assemblages.

Further east in the North Caucasus, the unequivocal presence of MP hominins, from at least MIS 5e or earlier to middle MIS 3, was based until recently on the archaeological record from only one stratified MP site—Weasel cave, located in a tributary valley of the Terek River in the eastern part (Kazbek Mt. region) of the north-central Caucasus (Fig 1). The cave is excavated by N. Hidjrati from 1981–present, but the results are published only preliminarily (Data 10 in S1 Text). As of 2010, 23 layers are assigned to the MP at Weasel cave, and containing Typical Mousterian or Denticulate Mousterian with Levallois blade technology industries [13].

Based on the published data [14–16], Golovanova and Doronichev [8: 53] suggested that the Levallois laminar Mousterian assemblages from Weasel cave are primarily similar to the Levantine Mousterian and Zagros Mousterian assemblages, and later Golovanova [10] noted that the lithic assemblages from layers 12–14 in this cave, which date to MIS 3–5 [13], show a particular similarity to the Zagros Mousterian industry that Golovanova and Doronichev [7] defined earlier in the Lesser Caucasus and Armenian Highlands. The Zagros Mousterian assemblages are characterised by unifacial tools, with prevailing of various lateral side-scrapers and convergent tools (scrapers and points), the presence of truncated–faceted pieces, and laminar or Levallois recurrent flaking technology, with high indexes of prepared and faceted platforms on flakes.

The results of comparative lithic analyses allowed us to suggest that two culture-different Neanderthal populations inhabited the western and eastern halfs of the North Caucasus during the Late Pleistocene. However, more representative evidence was required to identify cultural peculiarities of MP assemblages in the north-eastern Caucasus and thus confirm this proposal.

In 2016, the first stratified MP site of Saradj-Chuko grotto was discovered in the Elbrus Mt. region, in western part of the north-central Caucasus [17, 18] (Fig 1). The results of 2017–2019 excavations and multidisciplinary research in this site [19] (Data 4–7 and 9 in S1 Text) indicate cultural and technological peculiarities, and differences in subsistence and lifeways between the Neanderthal groups that inhabited the western (Kuban River basin) and eastern (Terek River basin) halfs of the North Caucasus. Also, the findings of rare bifacial tools at Saradj-Chuko grotto allowed us to suggest contacts between these populations.

In this paper, we offer a synthesis of modern data on the MP Neanderthal occupation of the North Caucasus in the Late Pleistocene. Based on the results of a comparative analysis of MP lithic assemblages dating from MIS 5 to MIS 3, which were recovered in two cave sites—Mezmaiskaya cave in the north-western Caucasus and Saradj-Chuko grotto in the north-central Caucasus—and our reassessment of comparative data from other MP sites known in the north-eastern Caucasus, we offer a hypothesis that two culturally distinct populations of MP Neanderthals that had different origins inhabited the North Caucasus from MIS 5 to the end of Neanderthal history in MIS 3. Also, we discuss the archaeological evidence from Mezmaiskaya Cave and Saradj-Chuko Grotto that indicates contacts between these two culture diverse Neanderthal populations, and substantiate our conclusion about the character of these contacts.

## Materials and methods

### Materials

#### Mezmaiskaya cave

Mezmaiskaya cave is located 1310 m above sea level (asl), in the Sukhoi Kurdjips River valley, which is a small tributary of the Kurdjips River (itself a tributary of the Belaya River, Kuban River basin), about 50 km south of the city of Maikop, in the north-western Caucasus, Russia (Fig 1). Since 1987, when L. Golovanova started excavations on the site, about 80 m^2^ have been carefully excavated to a maximum depth of 5 m.

Mezmaiskaya cave preserves a finely layered sedimentary succession of Late Pleistocene and Holocene deposits (Data 2 and 3 in S1 Text). The basal Pleistocene strata (4–7)—excavated only in a test pit—contained no archaeological material. So far, six Holocene and 20 Late Pleistocene strata have been identified over the excavation area, including seven MP levels (2, 2A, 2B1, 2B2, 2B3, 2B4, and 3, from top to bottom) with ESR dates between ca. 70–40 ka BP [2, 10, 20]; six Upper Paleolithic and two Epipaleolithic levels dating to ca. 39–25 ka calibrated (cal) BP and 17.5–12.5 ka cal BP respectively [6]; and six post-Paleolithic levels dating from the Holocene.

#### Saradj-Chuko grotto

Saradj-Chuko grotto is located 935–940 m asl, in the Saradj-Chuko (or Fanduko) River valley, which is a small left tributary of the Kishpek River (itself a tributary of the Baksan River, Terek River basin), about 70 km north-east of Mt. Elbrus, 6 km south of the town of Zayukovo, and about 20 km north-west of the city of Nalchik, in the Elbrus region of the north-central Caucasus, Russia (Fig 1; Data 4–7 in S1 Text).

Since 2016, when E. Doronicheva started excavations on the site, about 46 m^2^ have been carefully excavated in 2017–2019 [18, 19]. The cave stratigraphic sequence is about 1–1.5 m thick. The dense basal deposit (Layer 7) is archaeologically sterile and composed mainly of ignimbrite and tuff slabs and fine-grained sediments. The overlying layers 6B–3 contain MP artifacts and animal fossils. The MP sequence is capped by Layer 2 with rare artifacts and Holocene deposits (layers 1, 1A–1C), with no evidence of Upper Paleolithic (UP) occupation.

### Methods

In both Mezmaiskaya cave and Saradj-Chuko grotto, a large-scale quadrant grid with 1×1m divisions was established to cover all areas of the cave sites, and one concrete datum point was emplaced. All material recovered *in situ* was piece-plotted in three dimensions, and a laser prism-based total station Nikon NPL-322 was used for 3D coordinate acquisition at the main datum. All excavated sediments were water-screened through nested 3mm and 1mm mesh.

The technological and typological characteristics of lithic artefacts from Mezmaiskaya cave and Saradj-Chuko grotto are based on detailed analyses of lithic assemblages (Data 1 and 9 in S1 Text). These studies were made by all authors. For the lithic analysis we employ a standard Palaeolithic typology using terms and definitions from Bordes [21] and Debénath and Dibble [22]. The data about other MP assemblages are derived from publications (Data 1, 9, 11, and 12 in S1 Text).

## Results

### The benchmark Eastern Micoquian sequence at Mezmaiskaya cave

The MP stratigraphic sequence at Mezmaiskaya cave, which includes seven layers, represents the most complete, longest and best dated benchmark sequence of the Eastern Micoquian in the north-western Caucasus. It shows a local development of this cultural tradition from its early stage (MIS 5) to the end of MP about 40 ka ago in MIS 3 (Data 2 in S1 Text). Mezmaiskaya cave has, for the MP period, unique preservation of organic material. This allowed a robust series of radiocarbon (on animal bones and charcoal) and electron spin resonance (ESR, on animal tooth enamel) dates [2, 20], which provided a background for assessing the chronological position of other Eastern Micoquian sites in the north-western Caucasus, which are undated or poor dated [12]. The combined accelerator mass spectrometry (AMS) radiocarbon and ESR dates indicate that the Eastern Micoquian Neanderthals occupied Mezmaiskaya and the entire north-western Caucasus in the range from over 70 ka BP_ESR/LU_ (Layer 3) to about 40 ka calBP (Layer 2)(Data 3 in S1 Text).

Fossil remains of three Neanderthal individuals have been found in two MP layers at Mezmaiskaya cave, in the top MP layer 2 (Mezmaiskaya 2) and in the lowermost MP layer 3 (Mezmaiskaya 1 and Mezmaiskaya 3), and detailed morphometric and palaeogenetic analyses have been performed for all individuals [23–28] (Data 3 in S1 Text). The ages obtained using branch shortening for Mezmaiskaya 1 and Mezmaiskaya 3 individuals are older than ESR estimates for layer 3, and indicate that these Neanderthals, representing the early Eastern Micoquian population in the north-western Caucasus, lived approximately 100–70 ka in MIS 5 [27, 28]. This older age estimate for layer 3 well agrees with the palaeogeographic and archaeological data from Mezmaiskaya and comparative data from other sites, indicating that the early stage of the Eastern Micoquian Neanderthal occupation of the north-western Caucasus corresponds to the interval from late MIS 5 through the end of MIS 4 [12].

Direct dating of Mezmaiskaya 2 Neanderthal from layer 2, representing the youngest dated Neanderthal specimen in the north-western Caucasus [27], produced an AMS ultrafiltered date of 39.700 ± 1.100 ^14^C BP [2]. This age estimate indicates that the Eastern Micoquian Neanderthals did not survive at Mezmaiskaya cave and in the whole region after ~39 ka cal BP.

### The Eastern Micoquian in the North Caucasus

The ‘Micoquian’, as defined by Bosinski [29] in Central Europe, or ‘Eastern Micoquian’, as defined by Gabori [30] in Eastern Europe, is the broadest and longest cultural complex produced by MP Neanderthals in West Eurasia at least from MIS 5 to the end of MP in MIS 3 [31]. Many authors describe the Micoquian also as the Keilmesser Group (KMG), assuming that asymmetric bifacial backed scraper-knives (*Keilmesser* in German) represent the ‘index fossil’ of this MP industry [32, 33]. Almost all stratified MP sites known in the north-western Caucasus were defined to represent a regional variant of the Eastern Micoquian industry produced by Neanderthals, which fossils were found at Mezmaiskaya Cave and several other MP sites in the region [7, 8, 10, 12, 34].

**Fig 2:**
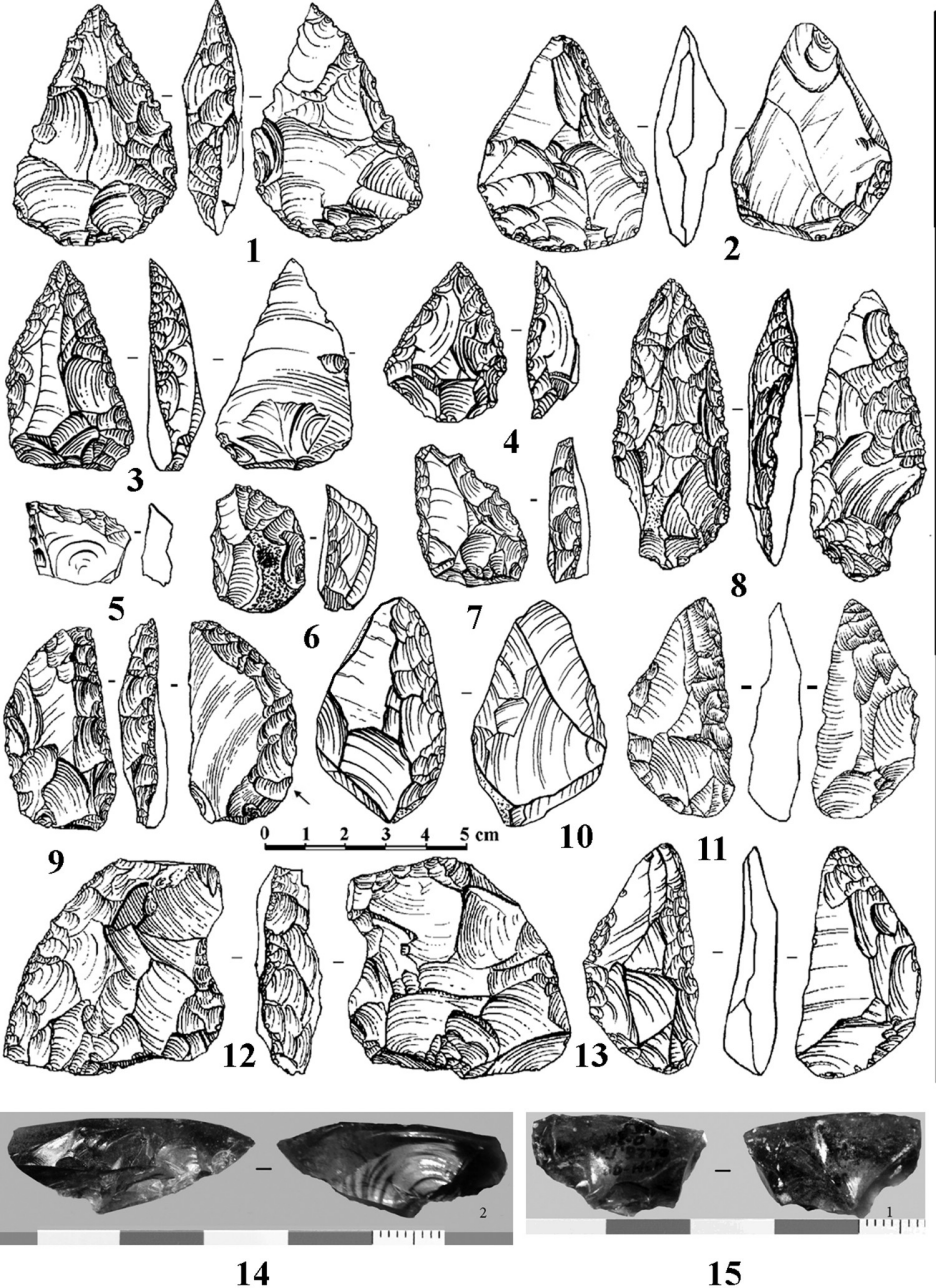
Mezmaiskaya cave. The retouched tools typical for the Eastern Micoquian (1–13) and small obsidian flakes found in layers 3 and 2B4 (14–15). 1, 2 –bifacial small handaxes; 3, 4 –Mousterian points; 5–7 –*déjeté* scrapers; 8 –bifacial leaf point; 9–13 –bifacial scraper-knives.

A non-Levallois and non-laminar recurrent flaking technology from non-prepared cores was defined in layers 3 and 2B4 in Mezmaiskaya Cave and is typical for other Eastern Micoquian assemblages in the north-western Caucasus. This technology resulted in the production of mostly short flakes, and was not oriented towards the production of Levallois or laminar blanks. In particular the earlier Eastern Micoquian assemblages from layers 3 and 2B4 at Mezmaiskaya, dating from MIS 5, are distinguished by the low indexes of blades (Ilam = 4–8), including laminar or bladey flakes, whose length (L) is between 1.5 and 2 times their width (m), following [22], and prepared (IF = 8–18) and faceted (IFs = 6–11) platforms. The earlier Eastern Micoquian assemblages also show a high percentage and large diversity of bifacial tools (for example, bifaces comprise 14.6% of total tools in layer 3 at Mezmaiskaya). In layers 3 and 2B4 at Mezmaiskaya, bifacial tools are represented by leaf-shaped bifacial points, triangular small handaxes, and various and numerous bifacial and partial bifacial scrapers and asymmetric backed scraper-knives (Fig 2). The later Eastern Micoquian assemblages, dating from MIS 3, show the increase in laminar products and the percentage of tools made on laminar blanks, and the decrease in bifacial tools, while similar tools with partial bifacial retouch are frequent in both early and late Eastern Micoquian assemblages in the region (Data 1 in S1 Text).

The presence of specific types of bifacial tools is the main feature that identifies the Eastern Micoquian sites in the north-western Caucasus and distinguishes them from other MP cultural contexts in the Caucasus. These specific bifacial tools include broad triangular small handaxes, leaf-shaped projectile points, various bifacial and partly bifacial convergent tools and side-scrapers, and asymmetric backed bifacial scraper-knives. These bifacial and partial bifacial tools provide the most striking feature of the Micoquian assemblages in the north-western Caucasus [11]. However, the ‘core’ of the Eastern Micoquian industry in this region are simple side-scrapers, which comprise about 20–40% of the total tools, and convergent tools (including Mousterian points, convergent and *déjeté* scrapers, and rare limaces), which comprise from 12% to 50%, but typically between 20–30% of the total tools. Transverse, diagonal and double scrapers are rare, and truncated-faceted points and scrapers are absent in all Micoquian assemblages in the region. A characteristic feature of the north-western Caucasus Micoquian industry is also the presence of bone retouchers that are unknown in other MP industries in the Caucasus.

### The benchmark Zagros Mousterian sequence at Saradj-Chuko grotto

The MP stratigraphic sequence of the Saradj-Chuco grotto, which includes three MP layers (3, 6A and 6B, from top to bottom) and was multidisciplinary investigated recently [17–19] (Data 4–7 in S1 Text) provides the background for defining cultural specificity of other MP sites dating from MIS 5 to MIS 3 in the eastern North Caucasus. Optically stimulated luminescence (OSL) dates indicate that the lower MP layer 6B was deposited during MIS 5, between about 90/80 and about 70 ka. Layer 6A was accumulated during early MIS 3, between about 60 and 50 ka. The end of the grotto occupation by MP hominins in Layer 3 is dated to about 45–40 ka. After a volcanic eruption recorded in layer 6A, MP hominins only occasionally visited Saradj-Chuko grotto, suggesting the volcanic activity crucially worsened living conditions in the Elbrus region [19, 35, 36].

Results of palynological and faunal analyses indicate that the main hominid occupation of Saradj-Chuko grotto in the lower part of layer 6B occurred during an interglacial phase, when the climate was warm and broad-leaved forests, with rare relict species currently growing in a subtropical climate (hickory, canadian hemlock, magnolia, and ephedra), grew in the area. Layer 6A was deposited during the period of a relatively warm (cooler than in layer 6B) and humid climate, characterized by broad-leaved forest and meadow-steppe vegetation. The upper MP layer 3 was accumulated during cold and humid climatic conditions compatible with meadow-forest or forest-steppe environments (Data 6 in S1 Text).

In Saradj-Chuko grotto, 90–98% of artefacts in all MP layers is made from a local obsidian originating from the Zayukovo (Baksan) obsidian source (Data 8 in S1 Text). This is the only MP obsidian industry in the North Caucasus.

**Fig 3:**
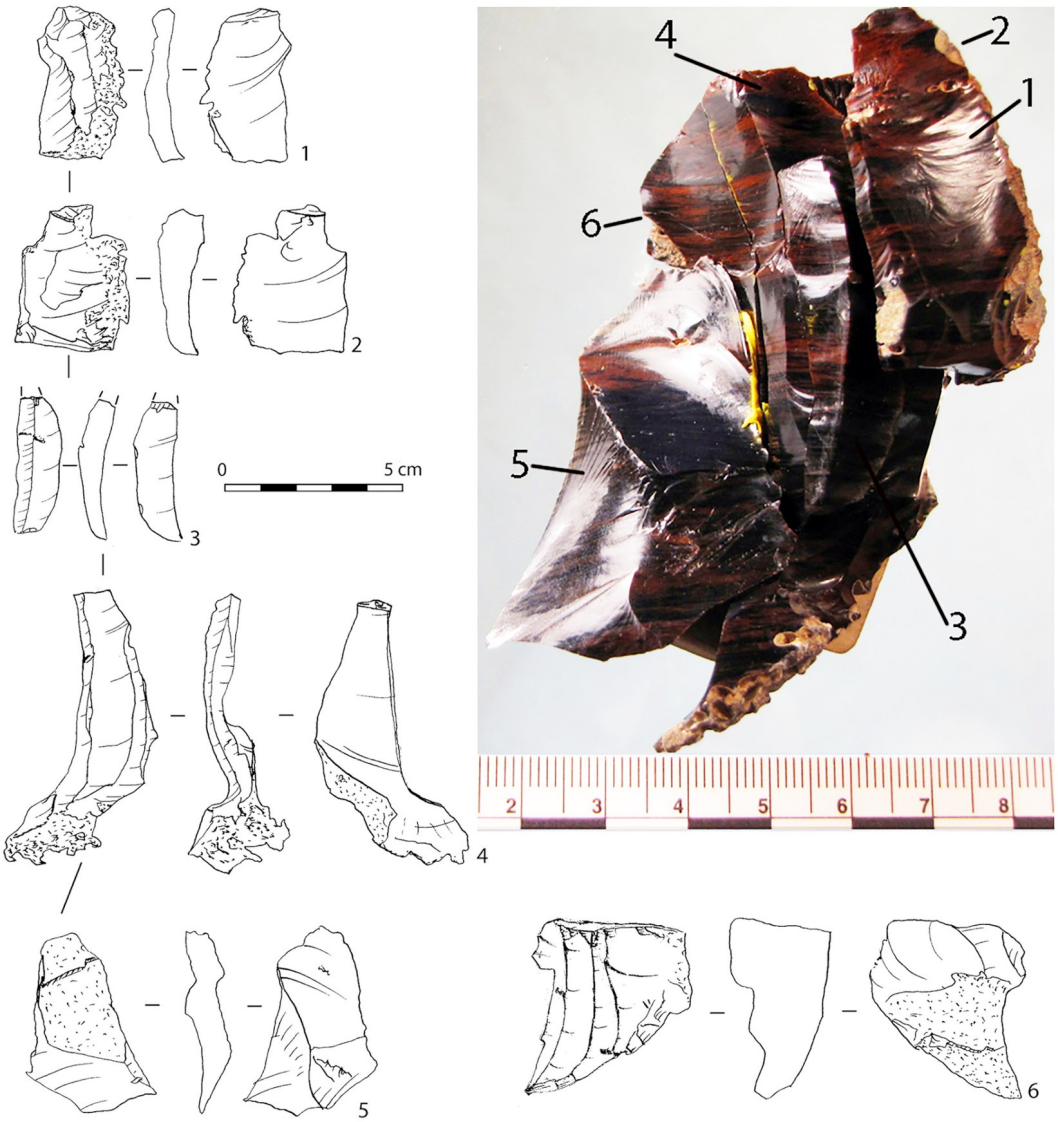
Saradj-Chuko grotto. 2017 excavation. Layer 6B. Drawing and photo of refitting of a one-platform core and five flakes, three of which are laminar flake (1) and blades (3, 4). Numbers indicate the sequence of removals.

The main MP assemblage from layer 6B includes 10,959 artefacts (2017–2019 excavations). In the assemblage, laminar or bladey flakes are abundant (27.2–45% of the total flakes). There are many flakes with prepared striking platforms (IF = 42.7), among which flakes with faceted platforms are numerous (IFs = 37) and dominate (IFs = 46–56) among laminar flakes. The technological indexes and refitting of an obsidian core and five flakes found within the main artefact concentration in layer 6B (Fig 3) indicate that laminar technology was used, and most laminar flakes were struck from prepared platforms. However, this was not the Levallois blade technology, because elongated blanks or blades (L ≥ 2 m) are rare (Ilam = 10.7), like Levallois blanks (IL = 12), and true blades and Levallois triangular flakes or points are very rare (5–5.5% and 1.3% of the total flakes, respectively).

**Fig 4:**
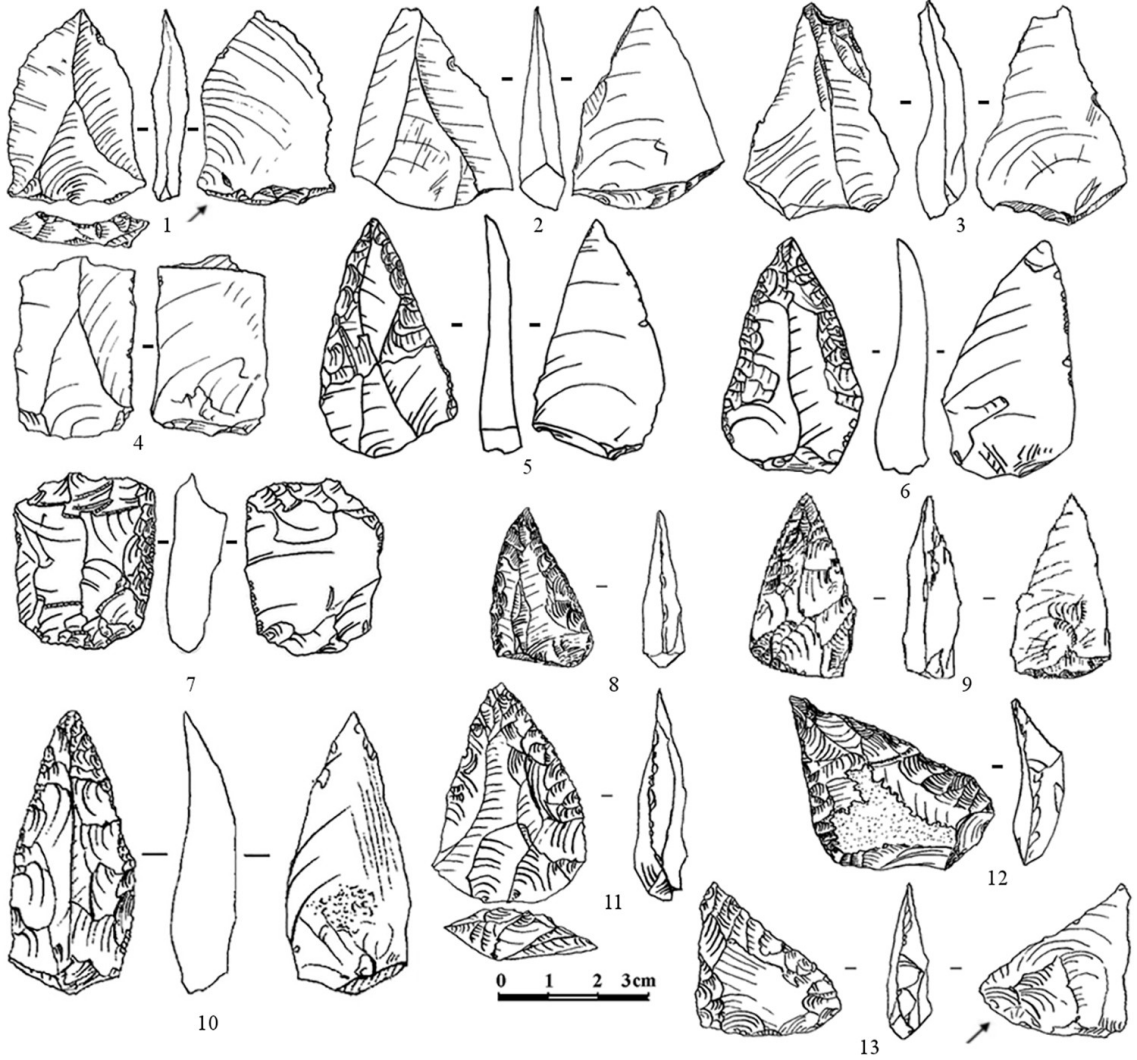
Saradj-Chiko grotto. The Levallois blanks (1–4) and retouched tools (5–13) typical of the Zagros Mousterian industry. 1–3 –Levallois triangular flakes (points); 4 –Levallois blade; 5, 6, 10 –elongated Mousterian points; 7 –truncated-faceted scraper; 8, 9, 11 –Mousterian points; 12, 13 –*déjeté* scrapers.

Among the 350 total tools from layer 6B, simple side-scrapers (31% of retouched tools) and various convergent tools (14% of retouched tools), which include *déjeté* points, *déjeté* scrapers, convergent scrapers, Mousterian points, and limaces, predominate. Also, there are 13 scrapers with ventral thinning or bifacial retouch, 12 atypical end-scrapers on flakes, five retouched Levallois points, two truncated–faceted pieces, and other tools (Fig 4; Data 9 in S1 Text).

The lithic assemblages from layers 6A and 3 are less numerous (610 and 80 artefacts respectively), but show the technological and typological features similar to the layer 6B assemblage, such as a high percentage of laminar flakes (35.7–45.1% of total flakes in layer 6A), and flakes with prepared and faceted platforms (IF = 45.5 and IFs = 38.8 in layer 6A), and rarity of blades (5.2% in layer 6A) and Levallois blanks. The 23 retouched tools from layer 6A include the tool types typical for the assemblage from layer 6B, mostly simple side-scrapers and convergent tools, and also show the presence of rare truncated–faceted pieces and atypical end-scrapers. Only a few retouched tools were found in layer 3, but they are similar to the tool types represented in layers 6B and 6A (Data 9 in S1 Text).

**Fig 5:**
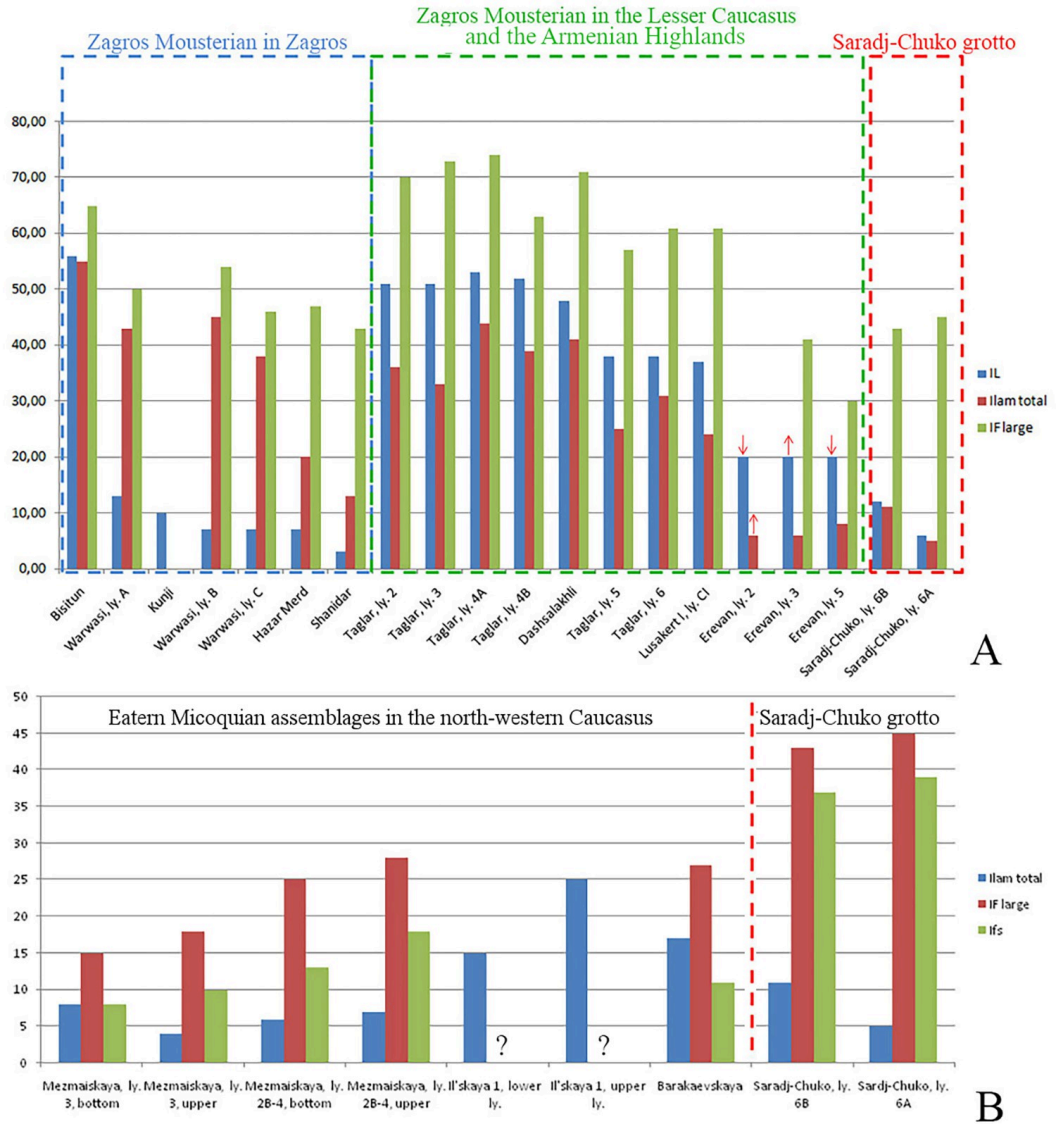
Comparison between the MP assemblages from layers 6B and 6A at Saradj-Chuko grotto and the Zagros Mousterian and Eastern Micoquian assemblages. A. Graph showing main technological indexes of the Zagros Mousterian assemblages in the Zagros, Lesser Caucasus and Armenian Highlands, in comparison to the assemblages from layers 6B and 6A at Saradj-Chuko grotto, in %. B. Graph showing main technological indexes of the Eastern Micoquian assemblages in the north-western Caucasus, in comparison to the assemblages from layers 6B and 6A in the Saradj-Chuko grotto, in %.

The results of recent research in Saradj-Chuko grotto indicate that the MP lithic assemblages recovered in this site represent a non-Levallois, but laminar and faceting Mousterian industry. The percentage of Levallois blanks (IL) and elongated flakes/blades (Ilam) are lower in the assemblages from Saradj-Chuko grotto than in the Zagros Mousterian assemblages in the Zagros, Lesser Caucasus and Armenian Highlands, while the high percentage of prepared platforms (IFl) in the Saradj-Chuko assemblages is similar to the Zagros Mousterian assemblages (Fig 5A). The laminar character of the knapping technology, indicated by the large number of laminar flakes (about 27–45% and about 36–45% of the total flakes in layers 6B and 6A respectively), and especially the high indexes of prepared (IFl) and faceted (IFs) platforms (Fig 5B) differ the assemblages from Saradj-Chuko grotto from the Eastern Micoquian assemblages in the north-western Caucasus. The predominance of unifacial tools, many of which are made on laminar blanks, and the presence of truncated–faceted scrapers, which are typical for Zagros Mousterian but absent in the Eastern Micoquian, are the typological features also specifying a cultural similarity of the MP assemblages from Saradj-Chuko with the Zagros Mousterian industry.

### Other Zagros Mousterian sites in the North Caucasus

Other stratified MP sites known in the east of North Caucasus, in which MP artefacts were found *in situ*, include the Weasel Cave in the Terek River basin, and Tinit-1 and Darvagchai-Zaliv-1 open-air sites, located in the Caspian Sea coastal region in Dagestan (Fig 1; Data 10 in S1 Text).

In Weasel cave, which is excavated by N. Hidjrati from 1981–present, 23 MP layers containing Typical Mousterian or Denticulate Mousterian industries with Levallois blade technology were identified [13, 15, 16]. Based on pollen and faunal data, the upper MP layers 4–11 are correlated with MIS 3, and layers 12 and 13 are correlated with MIS 4 –MIS 5c. Layer 14 may be also correlated with late MIS 5, while the lower layers 15–23 have an older age, but rare lithic artefacts containing no diagnostic Mousterian tools were found in these layers.

**Fig 6:**
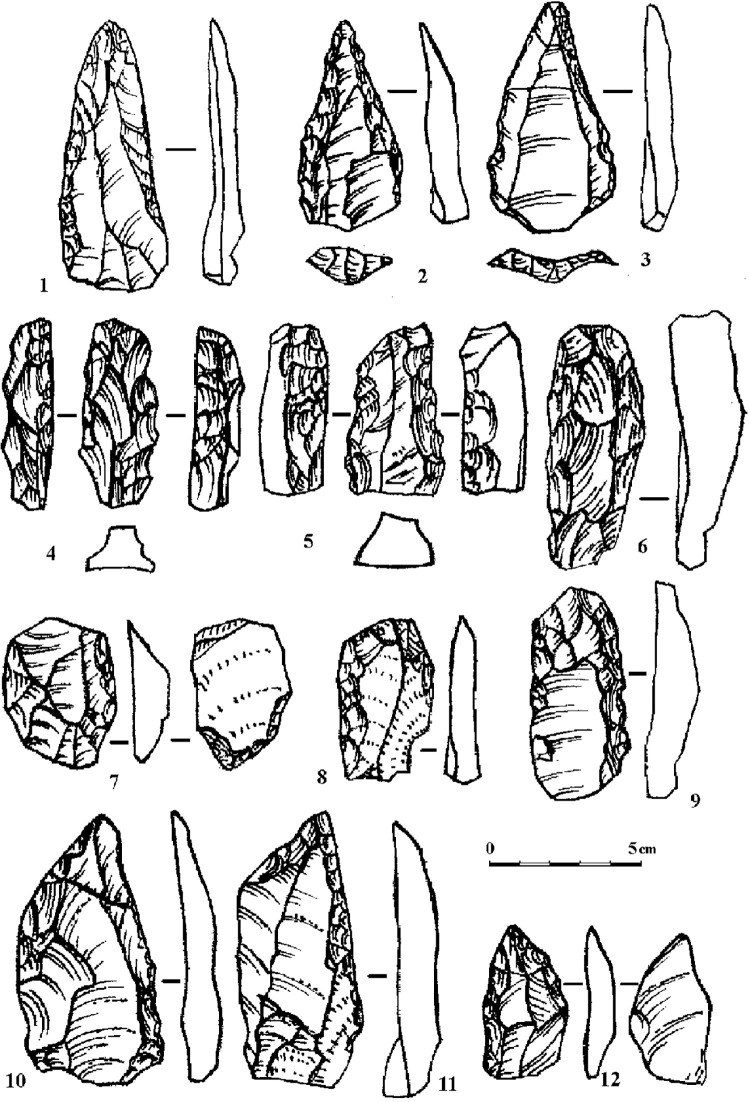
Weasel cave. The retouched tools typical of the Zagros Mousterian industry. 1, 2 –elongated Mousterian points; 3 –Levallois retouched point; 4–6 –thick double scrapers; 7 –truncated-faceted scraper; 8, 9 –side-scrapers; 10, 11 –side-scrapers made on Levallois blades; 12 –*déjeté* scraper. Modified from [8: fig 10].

Both the Levallois/laminar technology and the absence of bifacial tools typical for Eastern Micoquian distinguish the MP assemblages from Weasel cave from the Eastern Micoquian assemblages of the north-western Caucasus. Earlier, Golovanova and Doronichev [8, 10] noted a particular similarity of MP assemblages from Weasel cave (especially from layers 12–14, dating from MIS 5) with the Zagros Mousterian industry in the Lesser Caucasus and Armenian Highlands [7] (Data 11 in S1 Text). The features that make the MP assemblages from the Weasel cave similar to Zagros Mousterian are the Levallois/laminar technology and the tool set that includes tool types typical for Zagros Mousterian assemblages in the Lesser Caucasus and Armenian Highlands (Fig 6; Data 12 in S1 Text). The early MP industry from Weasel cave is characterized with laminar and Levallois blanks, including rare Levallois blades and points, as well as a high number of tools made on laminar flakes and blades. The tool set is characterized by a high percentage of convergent tools that include Mousterian and retouched Levallois points, *déjeté* scrapers, and other convergent tools. Also, narrow and thick bar-like double scrapers (so called “rods”) and truncated-faceted scrapers were found.

The Darvagchai-Zaliv-1 and Darvagchay-Zaliv-4 sites in Dagestan are dated from early MIS 5 (probably MIS 5e). Both sites produced small lithic assemblages that represent mainly flint-knapping workshops, in which mostly exhausted cores, other knapping products (mainly flakes), and rare retouched tools were found. The lithic assemblages from the Darvagchai-Zaliv-1 and Darvagchay-Zaliv-4 sites differ from the Eastern Micoquian assemblages in the north-western Caucasus for the complete absence of bifacial tools, while share technological similarity with all early MP industries with Levallois/laminar technology defined in the South Caucasus. However, the very small numbers of formal retouched tools found in both sites make it impossible to provide a reliable attribution of the lithic assemblages recovered in the Darvagchai-Zaliv-1 and Darvagchay-Zaliv-4 sites to any of the MP industries defined in the Caucasus [37] (Data 10 in S1 Text).

**Fig 7:**
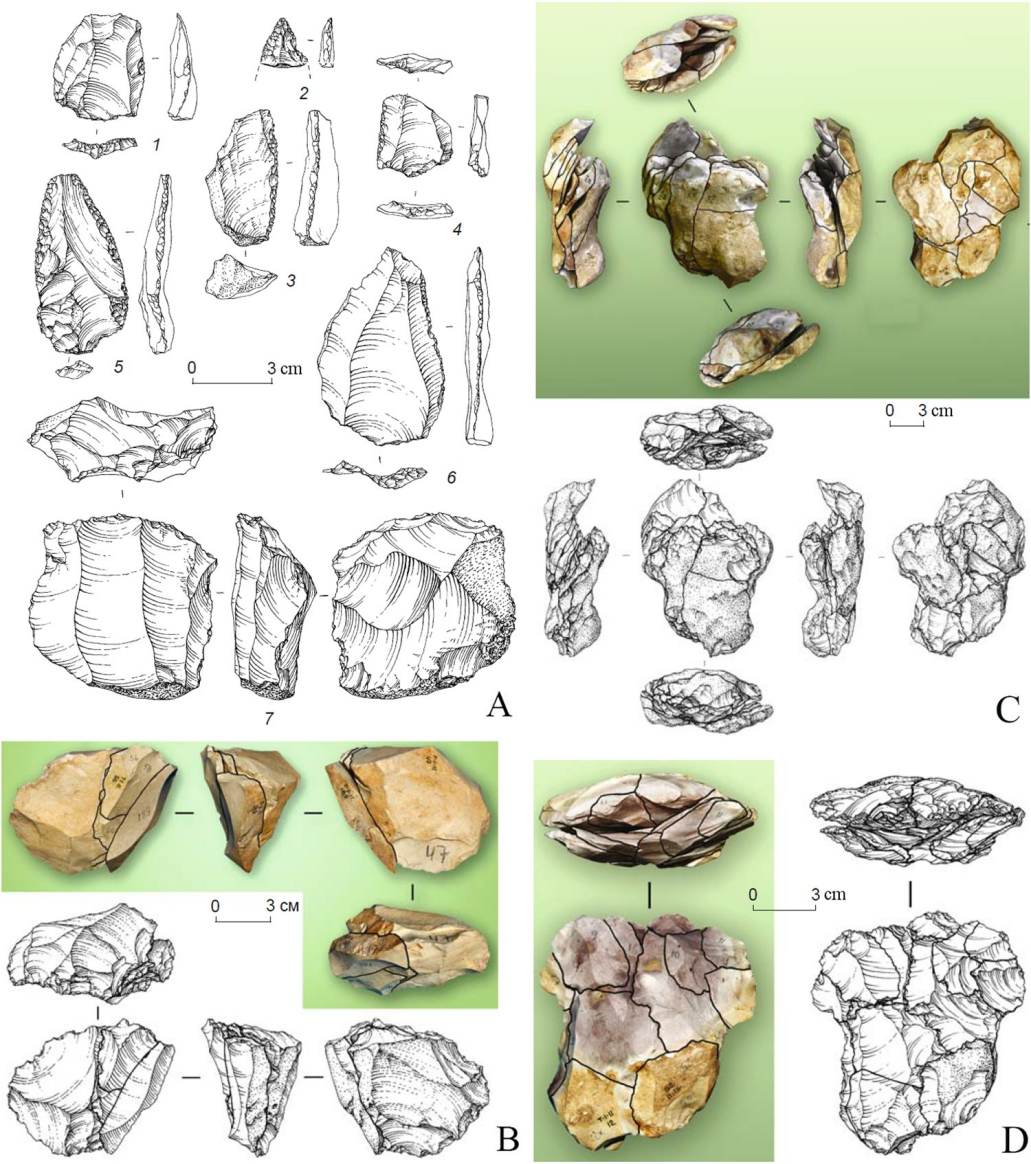
Tinit-1 open-air site. The Zagros Mousterian assemblage. A. The Levallois products and retouched tools (2–5) typical of the Zagros Mousterian industry. 1 –Levallois flake; 2 –a tip fragment of Mousterian point (?); 3, 4 –side-scrapers; 5 –elongated Mousterian point with a broken tip; 6 –Levallois triangular flake (point); 7 –Levallois recurrent core. B–D. Drawings and photos of three refitting samples, representing volumetric parallel flaking (B) and Levallois recurrent flaking (C, D). Modified from [38: figs 6, 8, 9, 11].

The Tinit-1 open-air site in the Rubas River valley, excavated in 2007–2010 and 2011, shows a finely stratified succession of 8 or 11 MP horizons that are dated from late MP, basing on average radiocarbon AMS ages between 43 and 51 ka calBP. Large lithic assemblages that were excavated in Tinit-1 (over 1600 lithic artefacts in 2007–2010 excavations and 335 lithic artefacts in 2011 excavation) show that this site represents a short-term stone knapping workshop and, probably, hunting camp. The excavators note that the stone knapping technology at Tinit-1 was aimed to the production of elongated blanks using volumetric parallel flaking and Levallois recurrent flaking methods [38]. In the upper horizons 1–4 and the lower horizons 5–9, the average blade percentage (Ilam) is 21% and 17.2% respectively, but increases to 29% and 31% respectively if laminar flakes are included. Most flakes (65.2% in horizons 1–4 and 62.2% in horizons 5–9) have plain striking platforms, while faceted and dihedral platforms are less represented (IF = 17 and IFs = 8.7 in horizons 1–4, and 15 and 11.2 respectively in horizons 5–9). Also, several refitting samples of a series of flakes (Fig 7C and 7D) or a core and flakes (Fig 7B) indicate that the flaking technology in Tinit-1 is similar to that represented by the refitting sample in the Saradj-Chuko grotto. The typologically definable retouched tools are few and comprise single and double side-scrapers, scraper-knives, notches, Mousterian and retouched Levallois points, atypical endscrapers, burin, one truncated-faceted implement, and a few other tools (Fig 7A; Data 10 in S1 Text).

The MP assemblages from the Tinit-1 site show many features of similarity, including in laminar technology and the tool types typical for Zagros Mousterian, with the assemblages from Saradj-Chuko grotto and Weasel cave, as well as the Zagros Mousterian assemblages from the Lesser Caucasus and Armenian Highlands (Data 12 in S1 Text).

The combined evidence from three stratified MP sites known at present in the east of North Caucasus, including Weasel cave, Tinit-1 and Saradj-Chuko grotto, shows that they share common technological and typological features that demonstrate the highest similarity with the Zagros Mousterian industry in the Lesser Caucasus and Armenian Highlands, while distinguish the MP industry of eastern North Caucasus from the Eastern Micoquian industry represented in the north-western Caucasus. This evidence suggests that the late MP Neandertal population of the north-central and north-eastern Caucasus was culturally related with the Zagros Mousterian Neandertal population of the Lesser Caucasus, Armenian Highlands and Zagros Mountains, and that the late MP industry of the north-eastern Caucasus represents a variant of the Zagros Mousterian industry.

### Contacts between Micoquian and Zagros Mousterian Neandertals

The petroarchaeological studies of lithic raw material sources that we started in 2007 showed that local Neanderthals in Mezmaiskaya Cave and other Eastern Micoquian sites in the north-western Caucasus exploited a large diversity of flint sources, both local (<30 km from the site) and non-local (about 30–100 km from the site). The area of lithic raw materials procurement in this way outlines the habitation area of the Micoquian Neanderthal population in the north-western Caucasus, i.e. the territory which resources were well known and regularly exploited by this population [39] (Data 8 in S1 Text).

In addition, rare (0.1% of the total artefacts) small obsidian artefacts (flakes and chips) were found in layers 3 and 2B4 at Mezmaiskaya Cave (Fig 2: 14, 15). The obsidian sourcing analysis indicated that the obsidian artefacts originate from the Zayukovo (Baksan) obsidian source in the north-central Caucasus, located approximately 250 km south-east from Mezmaiskaya (Data 13 in S1 Text). It should be noted that in the entire Caucasus obsidian artefact transport over 200 linear km have only been observed in UP sites [6, 9]. The findings of obsidian artefacts in MP/Eastern Micoquian layers at Mezmaiskaya suggest a high mobility pattern of the Eastern Micoquian Neanderthals, which was probably the highest among the MP/Neanderthal entities defined in the Caucasus.

The extreme rarity of obsidian artefacts found at Mezmaiskaya indicates that these obsidian items entered the cave not as a result of regular lithic raw material procurement by local Neanderthals or their exchange with the Neanderthal population in the Zayukovo obsidian source area, but as exotic pieces made from unusual lithic raw material, probably as a result of movements of individual groups across the landscape. These findings suggest that the Eastern Micoquian Neanderthals could sporadically reach the Elbrus region in the north-central Caucasus, and, consequently, have contacts with a local Neanderthal population that produced the Zagros Mousterian assemblages in the Saradj-Chuko grotto.

**Fig 8:**
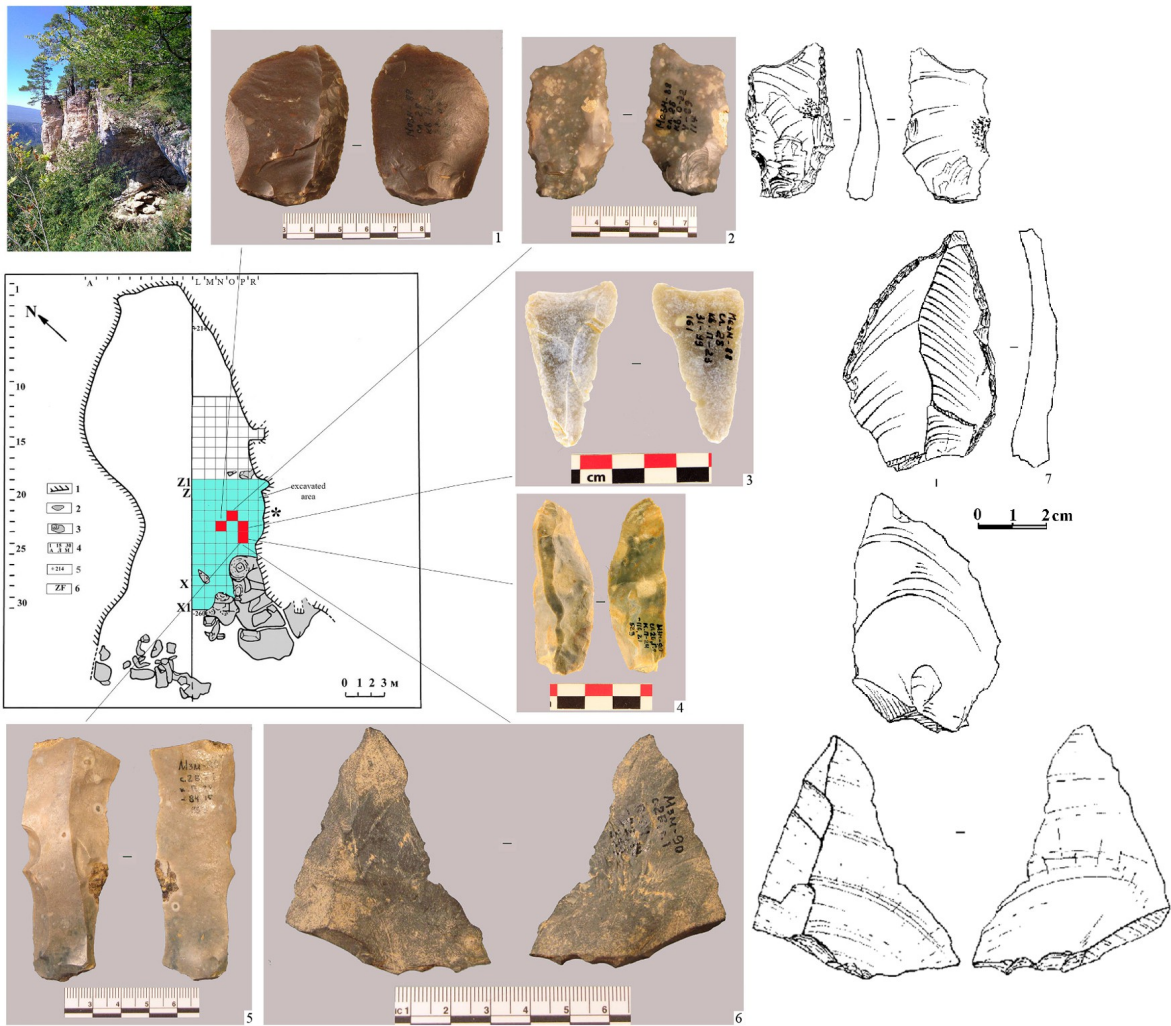
Mezmaiskaya cave. Photo of the cave, and plan of excavation showing the discovery points of Levallois artefacts (1–5, 7) atypical for the Eastern Micoquian industry and a flake made from exotic raw material, probably slate or volcanic rock (6), in Layer 2B4.

Earlier, we [10, 40] identified in Layer 2B4 at Mezmaiskaya cave a small artefact concentration that included several Levallois blanks and a flake made from exotic raw material, probably slate or volcanic rock (Fig 8). These artefacts and raw material are not characteristic for the Eastern Micoquian industry of the north-western Caucasus. We interpreted them as intrusive objects that likely entered to Mezmaiskaya cave from another cultural context, as we thought from the Khostinian Levallois-Mousterian cultural area in the Black Sea coast. However, the recent discovery of the Zagros Mousterian assemblages at the Saradj-Chuko grotto in the Elbrus region and the evidence of contacts of the Eastern Micoquian Neanderthals from the north-western Caucasus with the Zayukovo obsidian source located in the same region point to a different direction of cultural contacts, namely between culturally diverse Neanderthal groups that occupied the western and eastern halfs of the North Caucasus.

**Fig 9:**
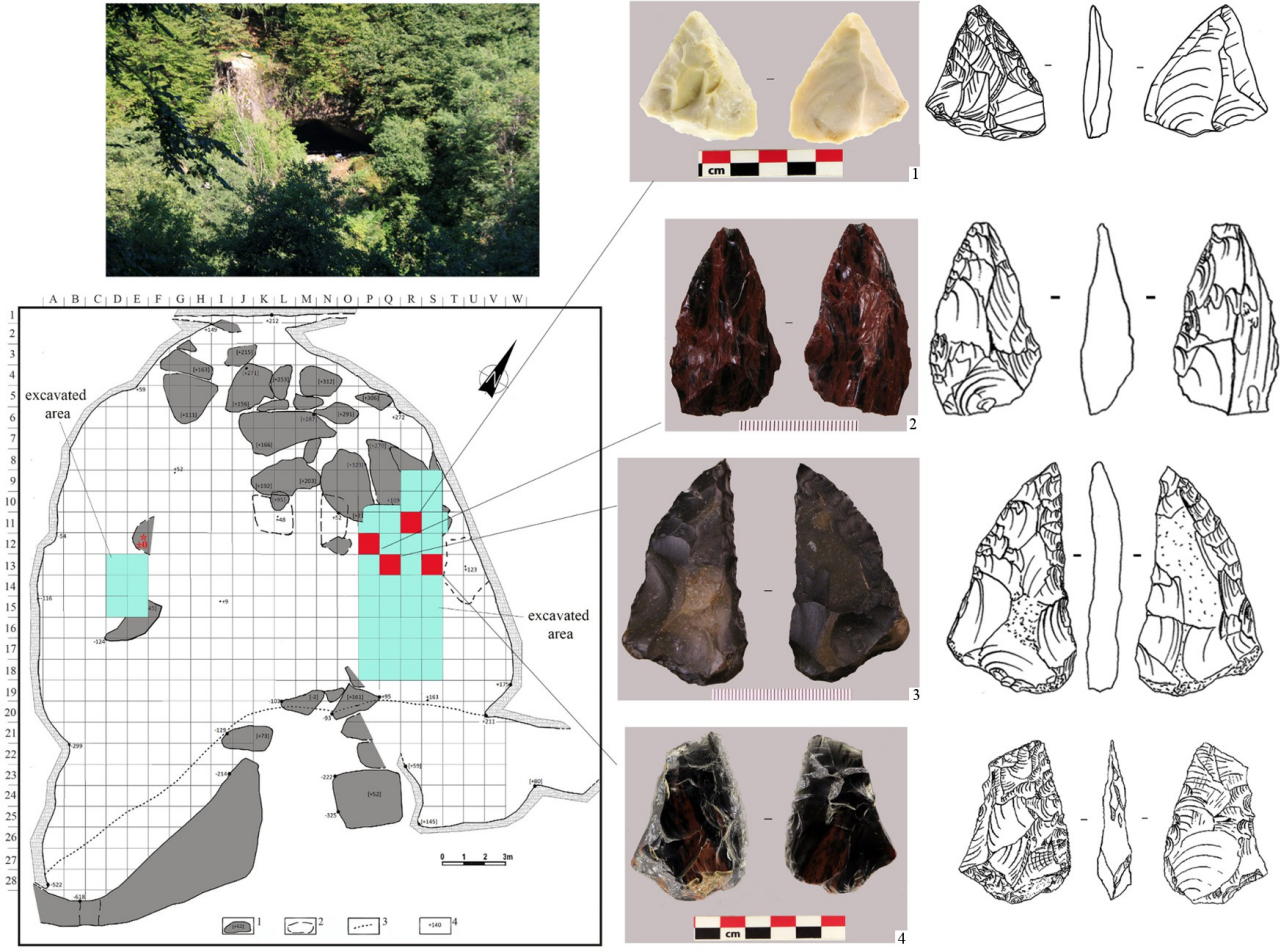
Saradj-Chuko grotto. Photo of the grotto, and plan of excavation showing the discovery points of artefacts typical of the Eastern Micoquian industry in Layer 6B. 1 –bifacial small handaxe; 2 –bifacial leaf point; 3, 4 –bifacial scraper-knives.

The evidence in support of contacts between the Neanderthal populations in the Elbrus region and in the north-western Caucasus was also found recently in Saradj-Chuko grotto. In layer 6B at Saradj-Chuko, there were found five bifacial tools that are not characteristic to Zagros Mousterian, but are typical to the Eastern Micoquian assemblages in the north-western Caucasus (Fig 9). All these tools are pointed instruments that could potentially served as a hunting mobile inventory, which hunters used for hunting animals or butchering hunting prey, and two of them were identified as a hunting projectile tip and a meat knife [19] (Data 13 in S1 Text).

## Discussion

In the Caucasus, including the North Caucasus, the state of studies on the Middle Palaeolithic period differs significantly between the western and eastern half of this region [7–9]. Research of the last five years confirmed the previous assumption [10] that two culturally distinct populations of MP Neanderthals, which had different origins and traditions of stone-knapping and tool-making, inhabited the North Caucasus from at least late MIS 5 to mid MIS 3. In the north-western Caucasus, almost all stratified late MP (dating from MIS 5 to mid MIS 3) sites represent a regional variant of the Eastern Micoquian industry, which was clearly produced by the Neanderthals (Fig 1; Data 1 in S1 text).

Previous studies indicated that a local Eastern Micoquian Neanderthal population in the north-western Caucasus, which inhabited this region from MIS 5 to MIS 3, was well adapted to various local environments, including cold and dry open steppes and subalpine meadows, forest-steppes, mountain mixed forest zone, and temperate broad-leaf forests [12]. During this long period, this Neanderthal population preserved a lineage of socially transmitted behaviour related to the manufacture of bifacial tools. The bifacial tools specific to the Eastern Micoquian stone-working tradition were found in various economic contexts: in flint-knapping camp-workshops, short-term hunting camps, and long-term and actively inhabited campsites [11].

This archaeological data are supported by the results of palaeogenetic analyses of there Neanderthal specimens from Mezmaiskaya cave [23, 27, 28]. They indicate that the Eastern Micoquian Neanderthals from the north-western Caucasus belong to European Neanderthals and represent a group of Neandertals that diverged from other European Neanderthals after about 110 ka [23] or approximately between 100–80 ka [27]. The palaeogenetic research also shows a great similarity between mtDNA genomes of three Neanderthals from geographically distant Eastern Micoquian contexts dated from late MIS 5—Stajnia S5000 from Stajnia cave (Poland), and Mezmaiskaya 1 and Mezmaiskaya 3—and they all fall beyond the mtDNA variation of the late European Neanderthals [28, 41]. Also, studies of Neandertal genomes from Mezmaiskaya indicate that there was a population turnover in the Eastern Micoquian Neandertal population of the north-western Caucasus during early MIS 3, most likely as the result of a population related to western European Neanderthals replacing earlier Eastern Micoquian Neanderthals in the region [27, 28].

The current archaeological evidence summarized in this article allows us to assume that a culturally different Neanderthal population, probably not related to European Neanderthals, entered the North Caucasus (along the Caspian Sea western coast) from the Lesser Caucasus and Armenian Highlands or more southern region in the Zagros mountains in Iran, and occupied the eastern part of the North Caucasus, as far west as the Elbrus region in the Terek River upper basin. The representative data from recent excavations at Saradj-Chuko grotto, which have yielded over 11,600 stone artifacts and numerous animal remains from three distinct MP layers dated from 92 to 41 ka ago [18, 19] (Data 5 in S1 Text), indicates that the Neanderthal population of the eastern part of North Caucasus had a distinctive technological tradition and the tool set that closely resemble those are typical to Zagros Mousterian in the Lesser Caucasus, Armenian Highlands and Zagros. This evidence suggests that the MP industry in the east of North Caucasus likely represents a variant of the Zagros Mousterian industry (Data 11 and 12 in S1 Text). The well dated lithic assemblages from the Saradj-Chuko grotto and comparative data from two other sites (Weasel cave and Tinit-1; Data 10 in S1 Text) also define that this distinctive Neanderthal population occupied the eastern part of North Caucasus from at least 90 ka to the end of Neanderthal history about 40 ka. Although Neanderthal fossils are not found yet within the Zagros Mousterian cultural area in the North Caucasus, only Neanderthal remains are associated with the distribution area of Zagros Mousterian from the Lesser Caucasus [42] to the Zagros mountains [43, 44].

In this regard, recent research identified also two possible routes for the Neanderthal dispersal from the Caucasus towards the east: a northern route from the Greater Caucasus via the northern Caspian corridor, associated with the Eastern Micoquian distribution to the Altai, and a southern route from the Lesser Caucasus via the southern Caspian corridor, associated with the spread of other Mousterian industries to Central Asia and Altai [45]. The researchers hypothesize that the two Neanderthal populations and cultural facies that could be associated with the southern route are the makers of the Zagros Mousterian and Levantine Mousterian.

The findings of rare small obsidian artefacts originating from the Zayukovo (Baksan) obsidian source in the Elbrus region in the Eastern Micoquian assemblages in Mezmaiskaya cave, as well as rare Levallois products not characteristic to Eastern Micoquian in Mezmaiskaya cave, and rare typical Eastern Micoquian bifacial tools likely representing a hunting mobile inventory of Neanderthals in Saradj-Chuko grotto suggest that small, mobile hunting groups of Eastern Micoquian Neanderthals from the north-western Caucasus could sporadically enter to a cultural area of the Zagros Mousterian Neanderthals in the eastern part of the North Caucasus. The current data suggests that the Eastern Micoquian Neanderthals were moving in the North Caucasus as far south-east as the Saradj-Chuko grotto area in the Elbrus region, located at the western boundary of the Zagros Mousterian distribution in the North Caucasus. The linear distances of obsidian artefact transport and moving of hunting groups (~250 linear km between Mezmaiskaya cave and Saradj-Chuko grotto) are consistent with those so far documented for MP Neanderthals in the South Caucasus and Armenian Highlands [5, 9].

## Conclusions

Basing on the current archaeological data about the MP in the North Caucasus, we propose that two culture different populations of Neanderthals settled the region from two various source regions: from eastern Europe along the Sea of Azov coast and from the Lesser Caucasus and Armenian Highlands along the Caspian Sea western coast (Fig 1). From late MIS 5 to mid MIS 3, a Neanderthal population bearing the Eastern Micoquian cultural tradition inhabited the western North Caucasus (Kaban River basin), whereas a Neanderthal population bearing the Zagros Mousterian cultural tradition inhabited the eastern North Caucasus (Terek River basin and the Caspian Sea northwestern coast). The archaeological data discussed above also suggests that irregular contacts existed between these two culture diverse Neanderthal populations in the North Caucasus. Our conclusions are based on the currently available data and need to be clarified by future studies.

## Supporting information

S1 Text Supplementary text. (DOC)
