# Supplementary material for: Archaeological evidence for two culture diverse Neanderthal populations in the North Caucasus and contacts between them
Source: PLoS One. 2023 Apr 13;18(4):e0284093. doi: 10.1371/journal.pone.0284093 (PMC10101426; doi:10.1371/journal.pone.0284093)
Supplement: S1 Text — (DOC) [file pone.0284093.s001.doc]

**Supporting information for**

**Archaeological evidence for two culture diverse Neanderthal populations in the North Caucasus and contacts between them**

Ekaterina V. Doronicheva^a,1^, Liubov V. Golovanova^a^, Vladimir B. Doronichev^a^,

Redzhep N. Kurbanov^b^

^a^ ANO Laboratory of Prehistory, 190020 St. Petersburg, Russia

^b^ Institute of Geography RAS, 119017 Moscow, Staromonetny per., 29-4, Russia

^1^ To whom correspondence may be addressed. Email: [edoronicheva87@yandex.ru](mailto:edoronicheva87@yandex.ru)

**This Supporting information includes:**

Supplementary text, Data 1 to 13

Supporting Information References 46 to 105

**Data 1. Eastern Micoquian in the north-western Caucasus**

In the North Caucasus, a major concentration of Middle Palaeolithic (MP) sites is known at present within the Northcaucasian mountain province in the north-western Caucasus, in the Kuban River basin, where about 80 MP sites are reported so far [7,8]. Most of them are surface locations and find spots, which only indicate possible human occupation during the MP period, but are very poor in artifacts and thus undiagnostic for a more detail lithic analysis and cultural identification. In the region, there are 12 stratified sites that produced in total about 30 occupational layers with MP assemblages, dating from MIS 5e (~130–120 ka) to middle MIS 3 (~40 ka) [9-12,34,39,46-52].

Almost all stratified MP sites known in the north-western Caucasus represent a north-western Caucasus (NWC) variant of the Eastern Micoquian industry, which was produced by Neanderthals [7,8,10,12,34]. The Eastern Micoquian sites are spread in this region from low foothills (Ilskaya-1 and Ilskaya-2, about 100 m asl) throughout middle mountain plateaus with elevations up to 1300–1500 m asl (Mezmaiskaya and Baranakha-4). They vary from open-air sites (Ilskaya-1, Ilskaya-2, Baranakha-4, Sredniy Khadjokh (Hadjoh), Khadjokh-2 (Hadjoh-2), and Besleneevskaya) to caves and rockshelters (Mezmaiskaya, Monasheskaya, Barakaevskaya, and Matuzka caves, and Gubs rockshelter 1) (Fig 1).

The MP sequence at Mezmaiskaya Cave represents the most complete and longest succession of Eastern Micoquian in the region, demonstrating a local development of this cultural tradition from its early stage throughout the end of MP in the north-western Caucasus. In Mezmaiskaya cave, the most robust series of radiometric (radiocarbon and ESR) dates for the regional Eastern Micoquian was obtained [2,20], which provided a background for assessing the chronological position of other, undated or poorly dated Eastern Micoquian sites in the region. Based on the MP sequence of Mezmaiskaya cave, it has become possible to link the regional MP assemblages into one developmental lineage of a local Micoquian tradition and identify temporal trends and changes in the regional Micoquian industry during a long period of time from late MIS 5 throughout MIS 3, between ~100–90 (based on mean genetic age ~89 ka and ~97 ka for Mezmaiskaya 1 and Mezmaiskaya 3 Neanderthal individuals respectively; [27,28]) and 40 ka ago [2,10]. Mezmaiskaya cave is also widely known as an MP site, in which three DNA analysed Neanderthal fossils, including the unique almost complete Neanderthal newborn skeleton (Mezmaiskaya 1), were found [23-28,53-56].

A feature of the NWC Eastern Micoquian industry are the characteristics of the knapping technology. A non-Levallois and non-laminar recurrent flaking from non-retouched platforms was defined in layers 3 and 2B-4 in Mezmaiskaya cave and is typical for other Eastern Micoquian assemblages in the north-western Caucasus [34]. The knapping technique used in these sites resulted in the production of mostly short flakes, and was not oriented towards the production of Levallois or laminar blanks. The earlier assemblages (from layers 3 and 2B4 in Mezmayskaya, Ilskaya-1, and Barakaevskaya) are distinguished by relatively low blade indexes (Ilam; 15.4–27.7), as well as indexes of prepared (IF; 14.6–27.5) and faceted (IFs; 6.4–10.6) striking platforms.

The presence of special types of bifacial tools is the main feature that identifies all the sites mentioned as the NWC Micoquian while distinguishing them from other MP sites in the Caucasus. These bifacial tools include small broad triangular handaxes (*breit dreieckige Faustkeilblätter*), laurel-leaf projectile points, various bifacial and partly bifacial convergent tools and side-scrapers, as well as asymmetric backed bifacial scraper-knives (similar to *Bocksteinmesser*, *Prondnikmesser, Wolgogradmesser* types; [29]). The earlier Eastern Micoquian assemblages in the north-western Caucasus differ not only in higher percentages of bifacial tools, but their larger diversity [7,8,10].

Although these bifacial and partial bifacial tools provide the most striking feature of the Eastern Micoquian assemblages in the north-western Caucasus, the ‘core’ of the industry are simple side-scrapers and typical Mousterian convergent tools (Mousterian points, *déjeté* points, convergent and *déjeté* scrapers, and rare limaces). The percent of convergent tools varies from 12 to 50%, but mainly between 20–30%, and the percent of simple side-scrapers is about 20–40%. Transverse, diagonal and double scrapers are rare. Various denticulate and notched tools, rare end-scrapers made on flakes and burins were also found in almost in all Micoquian assemblages in the region.

In the lower layers 3 and 2B4 in Mezmaiskaya Cave, there are leaf-like bifacial projectile points, small triangular handaxes, as well as various bifacial side-scrapers. Among the last, bifacial and partly bifacial backed scrapers or knives are the most numerous, some of them similar to *Bocksteinmesser*-type bifacial backed knives. Some bifacial scrapers resemble *Keilmesser* in having plano-convex trimming with one more carefully retouched lateral side and shaped base. There are also bifacial and partly bifacial knives similar to *Prondnikmesser*, bifacial and partly bifacial scrapers similar to *Wolgogradmesser*, and other partly bifacial convergent pieces [11]. In Ilskaya-1, there are leaf-like projectile points, small triangular handaxes, several types of convergent tools, fragments of triangular bifaces. Bifacial side-scrapers are represented by different types, including partly bifacial *Keilmesser*-like scrapers with well-trimmed lateral sides and bases, and *Prondnikmesser*-like tools with distal back thinned by elongated (pseudoburin) scars. There are also bifacial and partly bifacial side-scrapers similar to *Wolgogradmesser*, and other bifacial and partly bifacial side-scrapers. Some bifacial tools have contours like unifacial Chokurcha-type triangles. Many tools (convergent pieces, side-scrapers, and end-scrapers) have partial bifacial trimming.

Differences in the assemblage composition among the Eastern Micoquian sites in the north-western Caucasus are likely related to their various economic specialisation. The sites represents different types of occupations: flint knapping workshops (Hadjoh 2/layers 6 and 7, and Besleneevskaya), campsites with active on-site flint knapping (Monasheskaya and Barakaevskaya caves, Gubs rockshelter 1, and Baranakha-4), short-term hunter parkings (Matuzka, layer 4B), and active-habitation sites (Mezmaiskaya cave, layers 3 and 2B4).

Throughout the Eastern Micoquian occupation of the north-western Caucasus, this industry also shows a quite clear development, with a decrease in bifacial tools from the earlier to later sites [12: fig. 4]. In knapping technology, the Eastern Micoquian industry shows increase in laminar products and a number of tools made on blades in the later Micoquian levels at Mezmaiskaya cave [12: fig. 5]. While the later Micoquian assemblages in the north-western Caucasus differs from the earlier ones in lower percentages of bifacial tools, partlial bifacial trimming was used more frequently in the former. This is especially characteristic of the Barakaevskaya Cave assemblage [46], in which there are convergent tools and *déjeté* scrapers with ventral or dorsal thinning retouch, as well as simple side-scrapers with thinned backs. Bifacial tools include small triangular handaxes, a *Prondnikmesser*-like knife, tool similar to *Bocksteinmesser*, and several tools resembling Chokurcha-type triangles [7: figs. 10, 11, 14]. In the latest stage of the NWC Micoquian, bifacial tools are very rare. For example, in the upper layers 2 and 2A in Mezmaiskaya Cave, only a bifacial backed scraper and a triangular small biface were found. Also, ventral thinning is represented on diagonal and transverse scrapers in layer 2.

A characteristic feature of the NWC Micoquian industry, which distinguishes it from other MP industries in the Caucasus, is also the presence of numerous and variable bone artifacts. In Barakaevskaya cave, 109 bone retouchers made mainly from tubular bones of bison were identified [57]. A rich bone industry was found in Mermaiskaya cave, especially in the lower layers 3 and 2B4 [12: fig 3]. It includes numerous and diverse bone retouchers, bone flakes and chips, as well as a series of scrapers made by retouch on bone fragments, unfinished retouched bone point, and a unique bone projectile point tapered and shaped by scraping.

**Data 2. Mezmaiskaya Cave: MP stratigraphic sequence**

Mezmaiskaya Cave is formed in the Upper Jurassic dolomite limestone cliff about 20 m in height, and is situated about 100 m above the Kurdjips River level. It is more than 500 m^2^ (15–17 m in width and about 35 m in length), up to 10 m in height in the entrance (Fig 8), and faces southwest. In the interior of the cave, there is a chamber with a relatively horizontal floor while near the entrance the modern surface of the cave deposits is gently sloping outside. Mezmaiskaya Cave preserves a finely layered sedimentary succession of Late Pleistocene and Holocene deposits, which includes seven MP layers (2, 2A, 2B1, 2B2, 2B3, 2B4, and 3, from top to bottom):

Layer 2 – yellow-brown loam with a small amount of fine corroded limestone debris; more than 20 cm thick. This is the latest MP level.

Layer 2A – purple or violet-brown in colour loam with a small amount of smoothed and corroded limestone debris and rare calcite fragments; up to 40 cm thick.

Layers 2В1–2B4 are loams and sandy loams, with numerous fine corroded limestone debris, and rare larger limestone debris and large blocks. These layers are varying in shades of brown colour: 2B1 is light brown, 2B2 is darker brown, 2B3 is light brown, and 2B4 is dark brown.

Layer 3 – yellow loam, almost without limestone debris, with very rare calcite fragments; up to 90 cm thick.

Layer 3 is the oldest MP level, lying atop calcareous "floors" of the underlying stratum 4. The basal Pleistocene strata (4–7)—excavated only in a test pit—contained no archaeological material.

Stratum 1D, lying atop the MP sequence and immediately below the oldest Upper Paleolithic (UP) Layer 1C, contains volcanic ash, and no artifacts or bones. The MP strata are most completely preserved toward the interior of the cave. Near the cave entrance, heterogeneous erosive processes have destroyed MP and UP strata, and the Holocene strata unconformably overlie the MP deposits.

**Data 3. Mezmaiskaya Cave: chronology, environmental and faunal data for MP layers**

A series of 16 accelerator mass spectrometry (AMS) determinations was obtained in the Oxford Radiocarbon Accelerator Unit of the University of Oxford (Great Britain), using the most advanced ultrafiltration technique, on humanly cut-marked bones from MP layers, and the dates were combined into a Bayesian model [2]. The Bayesian modeling indicates that the majority of previous ^14^C results for the MP levels in Mezmaiskaya are either significant underestimates or lay beyond the current calibration limit, as well as that all radiocarbon estimates for the lower MP layers 2B3, 2B4, and 3 are clearly greater-than or near-background limit ages.

The results suggest that the majority of old and recent radiocarbon estimates for the MP sequence in Mezmaiskaya should be discarded, because they are beyond the radiocarbon limit age. Therefore, the age of the lower MP layers is consistent with the ESR chronology. The 14 ESR dates obtained for MP layers in Mezmaiskaya Cave do not depend significantly on uptake model (early uptake, EU, or linear uptake, LU) used, and they produce closely similar results within measurement errors [20]. The ESR dates determine the time interval of Neanderthal occupation of Mezmaiskaya Cave in the range from ~73 ka to ~40 ka ago.

For the lower MP layer 3, three ESR dates obtained on herbivore teeth from excavation horizons 3 and 4, which corresponds to the upper occupational level in this layer (data 4 in S1 Text), provide mean ages ranging from 60 to 73 ka BP_ESR/LU_ [20]. The lower occupational level in layer 3, which has an older age, can be dated approximately 85–100 ka, basing on mean ages obtained using branch shortening estimates from the mtDNA genome of Mezmaiskaya 1, which was found at the base of this layer [27].

According to multidisciplinary research in Mezmaiskaya Cave, the archaeologically sterile layer 4 corresponds to the opening of the cave by erosion [51,52,58]. It is worth noting that a conclusion about glacial conditions existed during formation of the main part of layer 4 was based only on a large amount of limestone debris noted by the researchers in the layer [59]. However, layer 4 actually contains rare limestone debris, but numerous fragments of calcite stalactites that fallen during the initial opening of the cave by erosion and aridification of the cave interior. Hominid occupation of Mezmaiskaya Cave started soon after it was completely opened and dried.

Multidisciplinary research indicates that a relatively mild (cool, but not cold) climate prevailed during the time of the lower MP layers 3 and 2B4, while a cold and dry climate, and sub-alpine meadows environment existed during the time of layer 2B3, which almost lacks archaeological finds [60]. The combined archaeological and pollen data, and ESR dates indicate that layers 3 and 2B4 correlate to late MIS 5, and layer 2B3 correlates to a cold MIS 4. During the upper MP layers 2B2, 2B1, 2A and 2 dated from MIS 3, Neandertals less intensive inhabited Mezmaiskaya cave, and the climate varied from cool and dry to cold and dry.

In the MP deposits in Mezmaiskya cave, two Neanderthal fossils have been found, namely, an almost complete Neanderthal newborn skeleton (Mezmaiskaya 1) that was found in the lowermost MP level (layer 3) and a juvenile (Mezmaiskaya 2) that was found in the uppermost MP level (layer 2). Detailed morphometric and palaeogenetic analyses have been performed for both individuals [23-28,53-56].

In Mezmaiskaya Cave, the earliest period of hominid occupation falls during the period of cool climate. At the time of the lowermost MP layer 3, the cave floor was covered by large limestone blocks, numerous stalactite fragments, and partly by calcite flows. Rodents inhabited woods and sub-alpine meadows are revealed in the lower MP layers 3 and 2B4 [59]. According to palinological data [51,52], grasses and bushes predominate in layers 3 and 2B4, while arboreal flora is xerophilous and poorly represented. In general, pollen spectrum suggests that the cave was located at that time in open grass environment near the tree line. Based on mean ESR estimates between 60–70 ka for layers 3 and 2B4 [20], mean genetic age ~89 ka for Mez1 Neanderthal individual [27], and taking into account the high elevation of the cave (1310 m asl), we propose that the initial Neanderthal occupation of Mezmaiskaya Cave has started during late MIS 5.

During the deposition of middle layers 2B3, 2B2, and 2B1, Mezmaiskaya Cave was less intensive inhabited by hominids, and sediments were repeatedly washed and destroyed. The previous radiocarbon dates suggested the MIS 3 age for layers 2B3 and 2B2 [7]. Later, Bayesian modeling indicated that the vast majority of previous ^14^C results for the MP levels are greater-than or near-background limit ages and should be discarded [2].

The age of layer 2B3 is determined by two ESR dates with mean ESR ages ranging from 52 to 59 ka [20]. Layer 2B3, which almost lacks archaeological finds, is characterized by a high pollen concentration, high herbaceous value dominated by *Asteraceae* (15–25%), *Cichoreaceae*, and *Rosaceae*, and high value of sub-alpine plants such as *Scabiosa*. The pollen spectrum indicates cold and dry climate conditions with sub-alpine meadows surrounding the cave [60]. The upper forest boundary was located lower than its current position. The combined archaeological and pollen data, and ESR dates suggest that layer 2B3 correlates to a cold period of MIS 4.

Layer 2B2 and the lower part of layer 2B1 are dominated by herbaceous pollen, and *Asteraceae* prevails (almost 50%) within this group. *Scabiosa*, a sub-alpine plant, and mesophilic herbs like *Cichoriaceae* (5–12%) and *Labiatae* are also represented. Tree pollen is poorly represented (15–20%). The total representation of *Picea*, *Abies, Pinus, Quercus*, and *Ulmus* is about 5%. This pollen spectrum may be attributed to a forest-steppe zone. In this period the cave was located near the upper boundary of the forest zone, or at the boundary of subalpine meadows and forest-steppe zone [60]. The climate was warmer and dryer than in the previous period, and likely cool and dry.

Тhe upper part of layer 2B1 and the lower part of layer 2A are characterized by a low pollen concentration. The spectrum shows maximum values of *Betula* and *Salix*, and increasing representation of *Poaceae*. During this period, the cave was surrounded by alpine meadows, and the climate was cold and dry.

Layer 2B1 was deposited when Mezmaiskaya cave was rarely visited by hominids that left in the layer only few artifacts. The study of layer 2B1 deposits using X-ray fluorescence (XRF) and X-ray diffraction (XRD) analyses, and infrared spectroscopy (IRS) indicated that this layer is probably an ancient soil and contains a volcanic ash of acidic (close to andesite-dacite) composition. This ash is similar in composition to andesidacite lavas of the Tash-Tebe volcano, located northwest of Mt. Elbrus, and dacite lavas in the Malka River valley, on the northern slope of Mt. Elbrus, which have ESR dates on quartz 39.0 ± 5.0 ka, and in the range from 45.3 ± 2.8 ka to 43.4 ± 9.3 ka respectively [61]. This data suggests that the eruption in the Elbrus volcanic province is the most likely source of volcanic ash in layer 2B1, which likely deposited in the interval between 48–42 ka ago [60].

Тhe upper part of Layer 2A and the top MP Layer 2 are characterized by a high pollen concentration and a high herbaceous representation with a predominance of *Asteraceae*. *Alnaster* and *Juglans* are represented among the rarer trees. Floral indicators suggest sub-alpine meadows. Climatic conditions were cool and wetter than in the previous period. Rodents typical of alpine and sub-alpine meadows are characteristic for these layers.

Several AMS dates indicate the age of the upper leyers 2 and 2A in the interval 40–46 ka cal BP [2], and two ESR dates determine a mean ESR age of 40–42 ka for Layer 2A and 37–41 ka for Layer 2 [20]. Direct dating of Mez2 Neanderthal from Layer 2 produced an AMS ultrafiltered date of 39.700 ± 1.100 ^14^C BP calibrated to 42.960–44.600 cal BP (68.2%), indicating that Neanderthals did not survive at Mezmaiskaya Cave after ~39 ka cal BP [2].

Bones from MP layers in Mezmaiskaya Cave exhibit the best preservation (typically Stage 0 or Stage 1, after [62]) six-stage sequence of weathering) in comparison to bones from other Eastern Micoquian sites in the north-western Caucasus that typically show a moderate (Stage 2) or moderately high (Stage 3) degree of weathering [63,64]. Ungulate remains exhibit the predominance steppe bison (*Bison priscus*), caprids (wild goat, *Capra caucasica* and sheep, *Ovis orientalis*) and less common red deer (*Cervus elaphus*). Within the Mezmaiskaya fauna, there are multiple indications that large carnivores brought a significant portion of the medium- to small-sized ungulate (sheep and goat) prey into the cave during the MP.

The distribution of bison and caprid remains suggests that the canivore contribution of caprids increased dramatically (contra decrease in bison primary contributed by hominids) toward the end of MP occupation when the cave was less frequently visited by the Neanderthals [60,64]. The deterioration of ecological conditions in the north-western Caucasus, documented by the pollen data and the deposition of volcanic ash in Layer 2B1 at Mezmaiskaya Cave, correlates with the lowest frequency of bison and the highest frequency of caprid remains within the sequence, together with the lowest densities of bones and artifacts [60: figs. 9, 10A], thus indicating that Neanderthal use of the cave fell to its lowest intensity [12].

**Data 4. Saradj-Chuko Grotto: MP stratigraphic sequence**

Saradj-Chuko grotto is located about 70 km north-east of Mt. Elbrus, 6 km south of the town of Zayukovo, and about 20 km north-west of the city of Nalchik (the capital of the Kabardino-Balkaria Republic, Russia). The grotto is formed in the Pliocene acidic volcanites (ignimbrite and tuffs), and sits in a deep (up to 200 m in depth) and forested valley of the Saradj-Chuko (or Fanduko) river (a small tributary of the Kishpek River, Baksan River basin), 26 m above the Saradj-Chuko river. Facing south-east, the grotto is over 300 m^2^ (up to 22 m in width and about 20 m in length from the drop line) and up to 6 m in height in the domelike entrance (Fig 9). The eastern part of the grotto is covered by large collapsed ignimbrite blocks that overlay the modern surface and are related to a partial and, apparently, one-time collapse of the grotto vault. Because these blocks overlap MP sediments, the latter have been preserved much better on this area than in areas closer to the entrance, where MP sediments were partially destroyed by erosion and human economic activity in historical times. Saradj-Chuko grotto was first excavated by E. Doronicheva in a small (1 × 1,5 m) test pit in 2016 and then excavated over a larger area in 2017–2019 and 2021 [17-19].

The geomorphological study indicates that Saradj-Chuko grotto is located in a peripheral part of the Chegem structural step, directly above the regional Nalchik flexure. The highest watersheds in this area—the Khara-Khora Mt. (1233 m asl) on the left bank of the Baksan river, and the watershed between the Baksan and Fanduko rivers, with altitudes up to 1100–1300 m asl—are composed of the Late Pliocene – Early Pleistocene (early Gelasian) volcanic rocks of the Lower Chegem volcanogenic formation, which is about 250–490 m thick. The volcanic rocks of the Lower Chegem formation cover watersheds of all local rivers, including the watershed between the Baksan and Fanduko rivers. The Saradj-Chuko River cuts thru laparite lavas, rhyolitic ignimbrites and tuffs of the Lower Chegem formation, which are replaced by Oligocene calcareous clays and marls downstream the river. The volcanogenic rocks of the Lower Chegem formation, which are exposed in the area, contain a number of dome-shaped structures that represent residues of extrusive domes (extrusions) formed in viscous lavas. Saradj-Chuko grotto is the largest of the extrusive domes in this area, and the grotto vault has a pronounced dome-like shape.

Saradj-Chuko grotto is a lava tube that was formed in volcanic rocks having a silicic composition (rhyolitic ignimbrites), in the lower (oldest) part of the Lower Chegem formation. This is the first lava tube cave in ignimbrites with MP deposits discovered in the Caucasus. According to geomorphological data, the beginning of the cave occupation by MP hominids can be estimated no earlier than 120–70 ka ago [19].

The Saradj-Chuko grotto stratigraphic sequence includes 11 lithological strata containing from modern to MP cultural deposits, as described below, from top to bottom. The upper layers 1, 1А, 1В, and 1С are Holocene deposits that have radiocarbon dates indicating the age of these layers from XIX to XVI century AD.

Layer 2 – yellow sandy loam, 11–24 cm thick. This is an almost archaeologically sterile latest Late Pleistocene stratum, which has yielded only several animal bones and stone artefacts.

Layer 3 – yellow sandy loam with thin levels of tuff gruss, 12–40 cm thick. This is the top MP layer, which has yielded several animal bones, and rare obsidian and flint artefacts, including an unfinished bifacial tool.

Layer 4 – grey-brown loam, 14–30 cm thick. The layer has yielded several animal bones and rare redeposited artefacts, but no *in situ* artefacts.

Layer 5 was initially defined as a separate stratigraphic level in the 2016 test pit, but later research has shown that this is a thin (5–7 cm thick) tectonic crack, running obliquely through layers 2–4 and filled by dark-brown, locally almost black humus sandy loam. Few small animal bones and only two small flake fragments were found in this layer.

Pits initiated from Holocene layers, including a large pit from Layer 1C, and holes of burrowing animals severely disturbed the Late Pleistocene layers 2–4 in some parts of the excavation located deeper into the cave, and roots of modern plants and drip water erosion disturbed these layers on lines 18 and 19 in the entrance part of the cave. Of the greatest interest is the lower layer 6 (defined in the 2016 test excavation), which was subdivided into two separate layers (6A and 6B) in the 2017–2019 excavations. Both layers have yielded abundant MP artifacts and fossilized animal bones, but only layer 6B was defined as a level of active Neanderthal occupation of the cave. In both MP layers 6A and 6B, some bones were partially replaced by secondary iron hydroxides, while some bones had decomposed to the condition of decalcified phosphate clusters. Both layers are lying with a slight inclination to the west.

Layer 6A – grey clay-rich sandy loam with rare tuff fragments, about 20–40 cm thick.

Layer 6B (main MP level) – dark brown to orangy-brown clay-rich sandy loam with rare small fragments and more rare larger blocks of ignimbrite and tuff, about 20–40 cm thick.

Layer 7 is the lowest stratum containing no artifacts or bones. The layer consists of numerous bar-shaped fragments of ignimbrite and tuff that are weakly cemented with iron hydroxides, which appear to make up the cave floor, and has a visible thickness up to 30 cm reached in the test pit.

**Data 5. Saradj-Chuko Grotto: radiocarbon and OSL dating, and sediment analyses**

In Saradj-Chuko grotto, one radiocarbon date 11.365 ± 120 yr BP (SPb-2537) obtained on bone from Layer 3 defines the age of the upper part of the Late Pleistocene sequence at about 13.274 ± 161 yr calBP. This age requires verification by other radiometric methods. All three radiocarbon dates on bone (Spb-2613 and Spb-2615) and charcoal (SPb-2616) obtained for MP Layer 6B are >40 ka BP, indicating that the age of this layer is beyond the limit of radiocarbon dating [19]. Several OSL dates, obtained more recently and reported for the first time in this article, indicate ages in the range from 36 to 82 ka ago for the MP layers in Saradj-Chuko grotto. Optically stimulated luminescence (OSL) dates indicate that the lower MP layer 6B is dated between 92 ± 5 and 82 ± 6 ka, while the upper part of this layer is dated to 78 ± 7 ka. The optical ages indicate that layer 6B was deposited during MIS 5. The MP layer 6A was accumulated between 60 ± 3 and 49 ± 3, which correlates with early MIS 3. The end of the grotto occupation by MP hominids is dated between 43 ± 3 and 41 ± 3 ka by two OSL dates for layer 4 and the uppermost MP layer 3.

In Saradj-Chuko grotto, 110 samples in total collected in 2018 from layers 1–7 were studied using the energy-dispersive X-ray spectroscopy (EDXS), and saturation isothermal remanent magnetization (SIRM) and quantitative X-ray diffraction (QXRD) phase analyses [18,19,36]. The low Irs values indicate breaks or slowdown in sedimentation of various duration due to changes of physics-chemical conditions (during these phases erosion of sediments inside the cave was insignificant due to a small humidity and the cave was dry), which may be related to climatic and environmental changes. Within the MP sequence, the most significant decreases in Irs values, suggesting the longer periods of the sedimentation break/slowdown, correspond to the boundary between MP layers 4 and 6A, and between MP layers 6A and 6B. The QXRD analysis indicated a high content of sodium and potassium chlorides in layer 6A, suggesting a relatively warm and humid climate.

The analysis of chemical composition and morphology of mineral particles indicated the presence of tephra (volcanic ash) in MP layers 6A and 4 [19,35,36,]. Melted grains of titanomagnetites and ilmenites found in layer 6А specify the presence of andesite-dacitic volcanic ash in this layer. The ash particles are similar in composition to pyroclastic materials of the dacite composition from both Elbrus volcano and Kazbek volcano. Saradj-Chuko grotto sits in the overlap zone of pyroclastic materials from both volcanoes. However, the OSL dating results indicated the OSL age of 49.1 ± 3 ka for layer 6A, which allows us to assume correlation of the volcanic ash from this layer with the eruption of the eastern cone of Elbrus, which ash has the dacitic composition and is dated > 30 ka ago [61]. After the volcanic eruption recorded in layer 6A, hominids only occasionally visited the cave. This suggests that the volcanic activity significantly affected the local climate and crucially worsened living conditions of hominids in the Elbrus region.

**Data 6. Saradj-Chuko Grotto: palynological and palaeontological data**

Results of palynological analyses define several stages of vegetation development in the Saradj-Chuko grotto area. The earliest stage, defined in the lowermost sterile Layer 7 and the lower level of MP layer 6B, is characterized by pollen zone I. This stage shows a diversity of tree and shrub species, suggesting interglacial age of these deposits. The angiosperm species include *Betula sect. Albae, Alnus glutinosa et incana, Carpinus caucasica, Carya ovata, Juglans regia, Juglans cinerea, Corylus colurna, Ostya carpinifolia, Ulmus campestris, Quercus aff. hartwissiana, Quercus ilex, Quercus pubescens, Fagus orientalis,* and *Castanea sativa*. Grassy vegetation is represented by single pollen of *Salsola soda*, *Compositae, Poaceaea, Apiaceae*, and *Urticaceae*. Among spores, representatives of *Polypodiaceae* predominates, including *Polypodium serratum* and *Cystopteris fragilis*, as well as single *Pteridium tauricum* (*Hypolepidaceae* family) was found.

The lower level of layer 6B is the oldest and most intensive MP occupational horizon in Saradj-Chuko grotto. The palynological data indicates that during that time broad-leaved hazel-hornbeam-oak forests with admixture of gray hazel and walnut, oriental beech, chestnut, and rare relict species currently growing in subtropical climate (hickory, canadian hemlock, magnolia, and ephedra) grew in the Fanduko River valley. Ferns (*Athyrium distantiifolium*, *Ophioglossum vulgatum*, and *Pteridium tauricum*) were widespread. The grass cover was poorly developed. *Chenopodiaceae* and motley grass occupied slopes of the valley, and *Salsola soda* grew in small saline areas in the river floodplain. The paleogeographic conditions identified for the lower part of layer 6B correspond to the interglacial climate, and the relict plant species that were found in this level are known in the North Caucasus only in MP sites dating before and during MIS 5, which suggests the correlation of main MP occupational horizon in layer 6B at Saradj-Chuko grotto with a warm phase of MIS 5 [19].

The middle level of layer 6B (above the main MP occupational horizon) is defined as pollen zone II, which is characterized by very low concentration of pollen and spores, and bad preservation of pollen grains in all samples. The features suggest a marketable cooling of the climate and decrease of summer temperatures. At that time, birch-elm and birch-alder forests, with admixture of oak, local thickets of hornbeam and hazel on slopes, and poorly developed herbaceous cover were widespread near Saradj-Chuko grotto.

Layer 6B yielded the richest faunal assemblage, in which more than 500 bones of large mammals adult individuals were identified. Remains Caucasian goat prevail (60%), followed by bison (13%), red deer (10%), and roe deer (10%), wild horse (*Equus caballus*; 5%), and other ungulates (<1% in the total) that include mountain sheep, wild boar and chamois. The predatory animals (1%) are represented by single bones of a forest cat (*Felis silvestris*), fox (*Vulpes vulpes*), and a small representative of the marten family (*Mustelidae*). Small mammals are also abundant and include remains of shrews, hares, marmots, ground squirrels, voles, and one tooth of hamster (*Cricetus sp*.). The identified species composition suggests that during the time of layer 6B the grotto was located near the border of the forest zone and high-mountain meadows, and the climate was moderately warm.

Three pollen zones (III, IV and V) were identified for the upper part of layer 6B and the main part of layer 6A. They seem to reflect two warm climatic stages (zones III and V), separated by a cooling stage (zone IV). At the early stage (zone III; upper part of layer 6B), moderately warm climatic conditions, and the development of hornbeam forests with admixture of bear walnut, oak and elm, with undergrowth from hazel were determined. The the later stage (zone V; layer 6A) is characterized by a warmer and wetter climate, and oak-elm-hornbeam forests with admixture of beech, linden, walnut, and bear walnut, and undergrowth from hazel, which were widespread inside the valley, and hornbeams growing on the lower parts of the valley slopes. The separating these warm phases zone IV (defined by one sample from the top of layer 6B) reflects the spread of sparse elm-lime forests, with admixture of oak and a developed herbaceous cover. In support of identifying zone IV as a separate environmental phase, analysis of the relative content of authigenic minerals in sediment samples indicates frequent short-term phases of a colder and humid climate during deposition of the upper part of layer 6B and layer 6A.

The analysis of fauna from layer 6A indicates a cooler climate than in the previous period. Among identified large mammals, Caucasian goat absolutely dominates (92%), while remains of other ungulates are rare, and include mainly isolated teeth of bison, wild boar, red deer, chamois, and roe deer. Small vertebrates are represented mainly by numerous teeth of voles, as well as rarer remains of hare, marmot, ground squirrel, and loir, and one tooth of mole rat were identified. Based on the predominance of remains of mountain (Caucasian) goat, one can assume that during that period the grotto was located in the subalpine meadows or montain steppes environment.

The study of remanent saturation magnetization revealed a sedimentation break/slowdown between MP layer 6A and layer 4, and the analysis of a relative content of authigenic minerals indicates a prolonged cooling in the upper part of layer 6A [19]. The palynological data suggests that the upper MP layers 4 and 3 accumulated mainly during cold and humid climatic conditions that correspond to pollen zones VI and VII, although the transition to more favorable climatic conditions is noted in the upper part of layer 3 (pollen subzone VIIIa).

In layer 4, artefacts were not found *in situ*, and it yielded a very small number of identifiable faunal remains, dominated by Caucasian goat. Although the insignificant number of identified large mammal species does not allow to unambiguously define climatic conditions during accumulation of this layer, the presence of both forest and subalpine species in layer 4 suggests mountain meadow-forest conditions and a moderately humid climate.

In the upper MP layer 3, Caucasian goat predominates (63%) among large mammals, followed by bison (25%). Remains of other ungulates, including wild boar, red deer, and chamois, are rare. Among predatory mammals, which account 6% of the total NISP in this layer, leopard (*Panthera pardus*) is determined. Also, numerous isolated teeth of voles and several teeth of gopher (*Citellus sp*.) that lives in mountain steppes with a dry climate were found. The faunal composition suggests that during accumulation of layer 3 the grotto was located near the border between forest zone and mountain meadows or steppes zone, with relatively dry climate.

**Data 7. Saradj-Chuko Grotto: microstratigraphy and planigraphy**

In Saradj-Chuko grotto, we performed analyses of microstratigraphy and space distribution of lithic artefacts in MP layer 6B [19], using the method described in [40,65]. The microstratigraphic analysis indicates that the main concentration of lithic artefacts in layer 6B coincides with the distribution of bones on most of the excavation area where this layer is about 20 cm thick. The artefacts and bones are especially concentrated in quadrant lines 9–12 inside the grotto, indicating that this part of the grotto was more convenient for hominid occupation. Almost all artefacts and bones found in layer 6 lied subhorizontally and according to a slight inclination of the bottom of layer 6B towards the grotto entrance, indicating both *in situ* position of artefacts and bones, and that the artefacts/bones concentration level in layer 6 represents a well-preserved archaeological horizon. All tools found in layer 6B are associated with the main concentration level. Only single artefacts (0.04% of the total artefacts) lied with a high inclination and obliquely relative to the main concentration level, probably as a result of trampling by hominids and animals. Also, rare bones and lithic artefacts were found in layer 6 above or below the main concentration level, which can be related either with postdepositional disturbances, or with visits by hominids to the grotto during the time before or after accumulation of the main artefacts/bones concentration level.

Towards the entrance to the grotto, the the main concentration level becomes firstly less concentrated (on quadrant lines 13–14; Fig 9) and then separates in two sub-levels (on quadrant lines 15–18): the lower, including mostly lithic artefacts and rare bones; and the upper, including mostly bones and rare lithic artefacts, and showing a more dispersed vertical distribution of artefacts and bones. This bifurcation can be interpreted as a result of that the main concentration level in layer 6B represents a palimpsest of at least two occupation episodes that were separated by a short time interval. Also, we see an increasing concentration of bones relative to artefacts towards the entrance to the grotto, starting on quadrant lines 16–18 and especially on lines 17–18. This may indicate that hominids could use this area in the entrance part of the grotto as a garbage zone, where they threw unused remains of animals consumed for food. It should be noted the composition of lithic artefacts found in quadrant lines 17–18 indicates that either stone knapping or tool production were not performed by hominids on this area.

The planigraphic analysis indicates that the artefact/bone concentration level in Layer 6B is related with two surface hearths that were found on quadrants R–S – 10–14 during the 2018–2019 excavation [19: figs 54–56]. Hearth No. 1 (on quadrants R–S – 12–14) has an oval shape (about 180 × 140 cm) and is about 5–7 cm in thickness. Hearth No. 2 (on quadrants R–S – 10–11) has a rounded shape (about 40 × 30 cm) and is about 2 cm in thickness. Both hearths exhibit generally good preservation, and show similar diagenetically altered internal stratigraphy lacking ash levels, and are similar in associated archaeological material. The black levels (BLs) in both hearths represent sedimentary substrates composed of a dark loam, which mainly contain scarce, scattered black particles representing unidentified wood charcoal fragments, rather than a distinct level of fuel residues. Human impact was not observed apart from the effect of burning that resulted in the BLs. The two hearths are stratigraphically associated with numerous unburned obsidian artefacts and frequent faunal remains, as well as rare burned artefacts and bones. Taking into account the aforementioned bifurcation of the main concentration level, each of these hearths likely can be associated with one of the two occupation episodes.

We performed the determination of the maximum firing temperature of these hearths, using the magnetic susceptibility method [66,67]. The samples of burnt sediment collected from both hearths were used for this analysis. The maximum firing temperature of the hearths was determined at the temperature range between ~530–600 °C.

Like layer 6B, layer 6A lies with a slight inclination towards the grotto entrance and contains much less artefacts and bones, which have scattered vertical distribution within the layer. The top MP layer 3 lies like the lower MP layers 6B and 6A, but it contains much fewer artefacts and bones that have scattered distribution within the layer.

**Data 8. Raw material analyses**

**Mezmaiskaya cave**

The petroarchaeological research of sources of lithic raw materials used in the MP assemblages in the NW Caucasus began in 2007 and resulted thus far in localization of more than 50 lithic raw material (mainly flint) sources, many of which were exploited during this period [39,68-72]. Symbol nomenclature was applied for consistency of data presentation on various flint sources, with sources designated by KR (*kremen'* in Russian, means flint) followed by a unique integer. The authors created and used a collection (*lithothèque* in French) of flint samples from all geological flint outcrops studied in the region for a comparative petroarchaeological analysis of various identifiable types of flints in archaeological assemblages.

These studies indicate that in Mezmaiskaya cave MP Neanderthals used a wide variety of lithic raw materials, including sedimentary (flint, chert, limestone, and siltstone), metamorphic (slate), and igneous (obsidian and granite) rocks. In the lower MP layers 3 and 2B4 (1987–2001 excavations), a local low-quality flint or chert from the Azish-Tau source (KR-1) is the most exploited material (48% and 59% of the total lithic artefacts respectively; [39: tables 3, 4]. The nearest outcrop of this flint was found only about 2 km from the cave. Based on the composition of artefacts made from KR-1 flint, including cores, core fragments, tested pieces of flint, core trimming elements (CTEs), cortical flakes, and shatters, we assume that knapping of this flint took place in the cave. However, a relatively small number of large fragments and cortical flakes made from KR-1 flint suggests that initial operations of testing and decortication of flint nodules occurred directly on KR-1 flint outcrops, likely on flint-knapping workshops that existed in these areas. The KR-1 flint is also the main lithic raw material (39% of the total tools) that was used for tool-making in layers 3 and 2B4.

Also, Neanderthals in Mezmaiskaya exploited non-local high-quality flints that originate from sources located about 30–100 km from the cave. Petroarchaeological analyses have identified several flint sources that were exploited during this period (see details in [39,70]): Unakoz (Unakozovskoye, KR-2; about 30 km from the cave), Shahan 2–4 (KR-9–10; 30–40 km from the cave), Meshoko (KR-47; 40–50 km from the cave), Gubs (KR-7; 40–50 km from the cave), Besleneevskaya 1–2 (KR-3–5; 50–60 km from the cave), and Ahmet-kaya 2–3 (KR-42–44; 80–110 km from the cave). Two of these sources were exploited most intensively, and almost a third of lithic artefacts in layers 3 and 2B4 is made from flint that originates from these sources: brown flint from the Shahan (KR-9–10) source area and colored flint from the Besleneevskaya (KR-3–5;) source area.

Three (0.1% of the total artefacts) obsidian artefacts (two retouched flakes and chip) were found in layers 3 (two pieces) and 2B4 (one piece) in Mezmaiskaya cave [39: tables 3, 4]. Two obsidian artefacts from layer 3 were analyzed using X-ray fluorescence (XRF) method. The results indicate that both obsidian artefacts originate from the Zayukovo (Baksan) source in the north-central Caucasus, located approximately 200–250 km south-east of the cave.

**Saradj-Chuko grotto**

The layer 6B assemblage (2017–2019 excavations) includes 10959 artefacts, the vast majority of which (96.7–98% in different excavation years) is made from obsidian, including all cores, technical and primary flakes. About 46,2% of obsidian flakes have cortex on their dorsal surfaces, indicating that obsidian cobbles or pebbles were brought to the cave and knapped on-site. Artefact refitting of one core and five flakes confirms on-site knapping of obsidian and that the area of obsidian knapping is related with the main concentration of artefacts on quadrants R–S – 10–14 inside the cave.

The XRF analysis of 19 artefacts indicated that all obsidian artefacts at Saradj-Chuko grotto were made exclusively on obsidian derived from the Zayukovo (Baksan) source, located near the town of Zayukovo, about 5–7 km of the grotto [19]. To understand the geology and geomorphology of obsidian-bearing deposits in the Zayukovo (Baksan) obsidian source area, we undertook field surveys in 2016–2018 that allowed us to identified four main obsidian outcrops, labeled Zayukovo 1–4. The XRF analysis of 39 obsidian samples collected in these outcrops indicates that, while Zayukovo obsidian varies highly in colour, all local obsidian has a single homogeneous composition, which differs from other obsidian sources known in the Southern and Lesser Caucasus. Therefore, all obsidian outcrops belong to a single source area. The Zayukovo obsidian is mainly uniform aphyric black or brownish-red (mahogany), sometimes black with rare sanidine phenocrysts (< 0.01 mm), banded black, and brownish-red by color. In thin section, the obsidian shows alternating brown, reddish-brown, and colourless bands, each subdivided into thinner bands. In the Zayukovo obsidian outcrops, obsidian occurs as pebbles and rarer cobbles (up to 20 cm in size) in deposits on high river terraces for about 10 km along the Baksan River. In the Zayukovo obsidian source, obsidian occurs only in the Baksan-ges formation, which contains thick strata of coarse alluvial and lacustrine deposits, up to 50–60 m in total thickness. The geomorphological study indicates that the Baksan-ges formation deposits accumulated in a paleolake that existed within the Zayukovo depression during the late Gelasian (early Lower Pleistocene).

Only about 2.0–2.9% artefacts (352 pieces) in the layer 6B assemblage (2017–2019 excavations) are made from flint and much more rarer other rock types. Unlike obsidian artefacts, flint artefacts include only chips, flakes, shatters and tools, and no cores or primary flakes. This composition of flint artefacts indicates that flint was brought to the cave as ready to use flakes and retouched tools. Petrographic and geochemical analyses indicate local sources of flint artefacts. The nearest identified sources of light grey good-quality flint (about 1.8% of the total artefacts) are the Hana-Haku-1 and Shtauchukua-1 sources, located in the Hana-Haku River and Shtauchukua River valleys (small tributaries to the Baksan River), about 5–7 km northwest from Saradj-Chuko grotto, while the nearest identified source of pink good-quality flint (about 0.5% of the total artefacts) is the Kamenka-1 source related with alluvial deposits of the Kamenka River, located about 7 km southeast from the grotto. Also, about 0.4% of the total artefacts are made from other rocks, such as silicified limestone, ignimbrite, slate, sandstone, and dark grey and black flint. Preliminary results indicate that the artefacts made from dark grey and black flint may originate from more distant outcrops located near the village of Bedyk in the Baksan River valley or from black flint sources identified in the Chegem River valley [19].

In general, the identification of lithic raw material sources indicates that the MP hominids in layer 6B at Saradj-Chuko grotto exploited almost exclusively the raw material sources sitting in the distance up to 7 km from the site. The prevailing obsidian was brought to the site as cobbles or pebbles that were knapped on-site, while the artefacts made from various types of flint or other rock types entered the cave as flakes or retouched tools.

In the upper MP layers 6A and 3, the geochemical and XRF analyses indicated the use of the same lithic raw materials: predominantly local obsidian (96% and 90% of the total artefacts respectively), grey and pink flint (3.8% in layer 6A and single artefacts in layer 3), and other rocks (0.2% in layer 6A and a single artefact from beige flint in layer 3).

**Data 9. Saradj-Chuko Grotto: MP lithic assemblages**

**Layer 6B**

A large lithic assemblage (10,959 artefacts in total) that was excavated in 2017–2019 from Layer 6B indicates that this layer represents the level of most intensive (in average about 295 lithic artefacts per m^2^) occupation of Saradj-Chuko grotto by MP hominids. The artefacts exhibit a good preservation, lack damage and fluvial abrasion, and only rare artefacts are weathered or patinated. The layer 6B assemblage includes rare cores (35; 0.8% of the total assemblage), numerous flakes (2234; 18.4%), shatters (4052; 42.5%) and chips (4286; 38.2%), and quite abundant retouched tools (350; 2.6%).

In the assemblage, more than 90% cores have small (4–9 cm) sizes and are heavily reduced. The core reduction analysis (CRA), using the method described in [73,74] shows that, despite the predominance of spherical or oval shaped obsidian pebbles in the Zayukovo obsidian source, the reduction and modification of cores in layer 6B followed the method of unifacial reduction from one surface, which is identified on almost 95% cores. The abundant one-platform cores (40% of total cores) indicate that core reduction often ended at this stage, while two-platform (40%) bipolar and orthogonal cores, and three-platform (13%) cores reflect a more prolonged reduction. Cores exhibit mainly unidirectional parallel scars struck from a flat or slightly convex flaking surface, and more rare a single scar. The predominance of unidirectional, parallel flaking on cores agrees with the morphology of flakes, among which almost 50% flakes have unidirectional, parallel removals on dorsal surfaces, while flakes showing orthogonal or opposite removal patterns on dorsal surfaces are more rare. The technological analysis also indicates that the flaking from cores performed mainly from beveled striking platforms, that were mainly prepared by several small scars. In addition, the traceological analysis determined the use of abrasive finishing of the striking platform edge. The results of both the technological analysis of knapping products and traceological analysis of some flakes suggest the use of the direct percussion flaking technique using a hammer made from a soft stone or horn.

The single case of refiting of one-platform core and five flakes (Fig 3) also shows that a sequential removal of pebble cortex occurred in the process of parallel flaking, i.e. the final decortication of cores occurred in the course of cores reduction. About 46,2% of obsidian flakes have pebble cortex areas on dorsal surfaces, which confirms this conclusion. Also, as the result of parallel flaking method not only the amount of pebble cortex consistently reduced on cores and flakes, but the produced flakes consistently acquired the morphology of laminar flakes and elongated blades. For example, on the mentioned refitting core, three out of five flakes are laminar flakes, and two of them are narrow blades.

Laminar flakes (or bladey flakes, whose length (L) is between 1.5 and 2 times their width (m), after [22]), are abundant (30–45% of the total flakes), although elongated flakes or blades (L ≥ 2 m) are rare (Ilam = 10.7) and true blades are very rare (5–5.5%) among total flakes. Levallois flakes also are rare (IL = 12) and typical Levallois triangular flakes or points are very rare (1.3% of the total flakes). Many flakes have prepared striking platforms (IF = 42.7), and many of them have faceted platforms (IFs = 37) that are made using fine retouching. Faceted platforms predominate (IFs = 46–56) on laminar flakes. These indexes are lower than those Bordes (1950, 1953) recognized as sufficient indicators of Levallois technique (IF > 45, IFs > 30, and IL > 20; IL = 5–20 means that Levallois products are present, but their number is not sufficiently high for clear identification of Levallois technique). This suggests that the layer 6B assemblage represents a non-Levallois industry, and the conclusion is confirmed by the technological analysis of cores. However, the high proportion of laminar flakes and the high index of faceted platforms (IFs) indicate that it is a laminar and faceting Mousterian industry.

According to the results of CRA, the stone knapping technology in layer 6B at Saradj-Chuko grotto cannot be defined as Levallois technology, because it does not show the application of methods for controlling the morphology of flakes or determining their shape. Although some cores do correspond to so-called "Levallois" morphology (after [75,76]), the study using CRA shows that all of them, together with most of other cores in this assemblage, demonstrate only the final stage of core reduction. The results of technological analysis of both cores and flakes indicate non-Lavallois, but laminar and faceting characteristics of the MP industry in this site.

Retouched tools (334 pieces) comprise 2.6% of the total assemblage [19]. The on-site obsidian knapping and numerous chips (4286; 38.2% of the total assemblage), many of which resulted from retouching, suggest that most tools were manufactured in the cave. Among 350 total tools, including unretouched Levallois points, following the Bordes [21] typology, simple side-scrapers prevail (118 pieces), followed by *déjeté* (12), double (10), convergent (10), transverse (7) side-scrapers, and side-scrapers with bifacial retouch (2). There are 25 Levallois and 5 retouched Levallois points, as well as 10 Mousterian and 5 elongated Mousterian points, and 3 limaces. Also, 12 atypical end-scrapers, 11 retouched notches and 87 denticulated tools, 24 flakes with fine retouch, 7 miscellaneous tools, and two bifacial foliates were identified. Among the 334 retouched tools we note the predominance of various flakes with fine irregular retouch (134 pieces) and single scrapers (105 pieces; 31.4% of retouched tools), including prevailing 93 simple scrapers, and 7 transverse and 5 diagonal scrapers. Also, there are 13 scrapers with thinning retouch, most of which have thinning retouch from dorsal surfaces, but two tools have ventral thinning retouch and one backed scraper has bifacial retouch. Also, two truncated–faceted scrapers made on blade fragments and having truncated–faceting elements on both ends were identified. The retouched tools with two convergent edges are numerous (47 pieces; 14.1% of retouched tools). They include *déjeté* points (15) and *déjeté* scrapers (14), 5 Mousterian points, 3 limaces, and 6 convergent and 4 thick convergent tools, three of which have bifacial retouching.

**Layer 6A**

The lithic assemblage (610 artefacts in total) includes only two exhausted cores, 210 shatters, 205 chips, and a relatively large number of flakes (170, excluding flake-tools). A large discrepancy between the number of cores and flakes suggests that many flakes were not produced on-site, but brought from elsewhere. Two CTEs are represented by platform preparation flakes. Most flakes (52.8%) have cortex areas on dorsal surfaces. Among flakes, laminar flakes are abundant (35.7% of the total flakes), although blades are very rare (5.2%). Many flakes (34.7 %) exhibit unidirectional parallel scars on dorsal surfaces, and also more rarer flakes with orthogonal and bidirectional dorsal patters are represented. Most flakes have prepared striking platforms (IF = 45.5), among which flakes with faceted platforms prevail (IFs = 38.8). Also, many flakes have plain (38.0%) and punctiform (13.2%) platforms, while cortex platforms are rare (3.3%). These technological characteristics indicate a highly developed technique of striking platforms preparation and the predominance of unidirectional parallel flaking. The 23 retouched tools in layer 6A include tool types typical for the Mousterian industry from layer 6B. About one third of total tools are tools with converging edges (8 pieces), including one complete Mousterian point, three distal fragments of Mousterian points, two *déjeté* points and two *déjeté* scrapers. Three of these tools have ventral thinning retouch. There are two truncated–faceted scrapers that are characteristic to the Zagros Mousterian. Other tools include 3 atypical end-scrapers and 6 flakes with fine retouch.

**Layer 3**

A small assemblage from this layer (80 pieces) includes one exhausted core, 35 flakes, 17 chips, 15 shatters, and 12 tools. The technological characteristics of the layer 3 assemblage are conditional and cannot be considered statistically reliable due to the small number of artefacts: flakes with unidirectional scars on dorsal surfaces prevail (24 pieces), but blades are rare (12.8% of total flakes, including flake-tools), and flakes with faceted platforms are not numerous (IFs = 16.7). Retouched tools include déjeté point with ventral thinning retouch on the base, déjeté scraper with dorsal thinning retouch on the base, distal fragment of pointed tool, 3 simple side-scrapers, single side-scraper with dorsal thinning retouch, transverse side-scraper, bifacial scraper with plano-convex trimming, end-scraper on retouched flake, and two flakes with fine retouch.

**Data 10. Other stratified MP sites in the north-central and north-eastern Caucasus**

**Weasel Cave**

Apart from several localities that yielded surface or redeposited artefact assemblages tentatively dated to the Middle Palaeolithic (or Mousterian) in Dagestan [77], Ingushetia and Northern Ossetia-Alania [78], the Weasel cave (Myshtulagty Lagat in Ossetian) is the only stratified Middle Palaeolithic (MP) site found thus far in the Northern Caucasus east of the Elbrus region, in the Terek River basin. Weasel cave, located 1125 m asl, in a tributary valley of the Terek River (Northern Ossetia-Alania Republic), was discovered by N. Hidjrati. He excavates the cave from 1981–present, but only preliminary reports are published [13-16]. The excavations revealed a stratigraphic sequence over 22 vertical meters, which comprises 36 distinct lithological strata, including over 10 volcanic ash horizons and about 40 Palaeolithic occupation horizons in 23 layers.

Hidjrati divided MP levels in two lithostratigraphic complexes: late MP in the upper complex (layers 4–11) and early MP in the middle complex (layers 12–21). As of 2010, it is reported that 23 MP layers containing Typical Mousterian or Denticulate Mousterian industries with Levallois blade technology are identified in Weasel cave [13]. However, small artefact assemblages almost lacking formal tools were found in layers 15–17, and very small artefact assemblages containing no typical Mousterian tools were found in the lower layers 19–21.

According to palynological data, the upper MP layers 4–11, which formed during a period when local environment varied from subalpine meadows to birch and fir dominated forests, indicating a fluctuation between interstadial and stadial conditions, are correlated with MIS 3. During the time of layers 12–14, the climate was warm and humid, and the prevailing environment was a deciduous forest with birch and chestnut mixed with hornbeam, oak, elm, walnut, and lime. Based on the pollen and faunal data, layers 12 and 13 are tentatively dated between 50–90 ka (MIS 4 – MIS 5c; [13]). Layer 14 may have a similar age (late MIS 5), based on the similarity of pollen spectra and fauna. Faunal remains are very well preserved in Weasel cave. Caucasian goat dominates in fauna from the upper MP layers 5–11, while deer and cave-bear so-dominate in fauna from MP layers 12–14.

In contrast, the pollen spectrum of layer 15 indicates an alpine meadow environment and a cold climate. Layers 16–18 comprise a single pollen complex, when pine and juniper forests prevailed in the cave vicinity. An archaeologically sterile stratum 18 is a volcanic ash horizon related to eruption of the Kazbek volcano, located about 15 km from the cave. Stratum 18 has an Ar^39^Ar^40^ date about 200 ka. A pollen spectrum from layer 19 indicates a dry and warm climate, and the spread of deciduous forests composed of elm, beech, walnut, hornbeam, and oak in the area.

**Darvagchai-Zaliv-1 and Darvagchai-Zaliv-4 open-air sites**

Until recently, the knowledge about the MP period in the Caspian region of Dagestan, in the southmost part of the north-eastern Caucasus, was based on quantitatively insignificant redeposited materials arbitrary attributed to MP from several surface localities discovered in the 1960s [79]. During the last 15 years, since 2003, a significant number (about 1400) of artefacts were found in pebble conglomerates and deluvial deposits, dated to Late Khazarian transgression of the Caspian Sea (ca. 200–100 ka), in several sites discovered along the Darvagchai and Rubas river valleys in coastal Dagestan (about 40–50 km direct distance from the Caspian Sea coast), including Rubas-1–5, Darvagchai-Zaliv-1, Chumus-Inic, and several others [80].

Anoikin [80] reported that these assemblages in general are characterized by the predominance of parallel unifacial flaking method, and radial and Levallois flaking methods are also present. These flaking methods were used to produce flakes and elongated blanks, including rare Levallois points, but the technological indices indicate a non-Levallois (IL < 20) and non-laminar (Ilam ~ 10) technology, with some presence of Levallois flaking and with low index of prepared platforms (IF < 20). Retouched tools are made on flakes and include prevailing side-scrapers, denticulates, notched tools, and perforators, abundant atypical end-scrapers and knifes, as well as rare Levallois and Mousterian points, limaces, and Upper Palaeolithic types (burins and borers). Truncated-faceted tools have not been identified. He concluded that these assemblages represent the early MP (probably MIS 5) stage of hominid occupation of the region, share cultural similarity with Zagros-Taurus Mousterian from the South Caucasus and Zagros, and likely represent a local variant of this Mousterian industry.

Among the recently discovered MP sites, the Darvagchai-Zaliv-1 open-air site in the Darvagchai River valley, excavated on a large area (>100 m^2^) in 2009–2017, represents one of the most significant early MP sites in the region that was multidisciplinary studied. Based on the results of research, the excavators [37,81] concluded that the MP assemblage from layer 3 in Darvagchai-Zaliv-1 correlates with MIS 5e, 110–125 ka ago (layer 3 is a paleosoil, in which the Blake palaeomagnetic excursion, 120–100 ka BP, was defined). In layer 3, artefacts occur mainly as isolated clusters, within which several artefact refits were found. The layer also contains numerous scattered wood charcoals, concentrated mainly in the lower part of the layer, where two disturbed hearths were found. The lithic assemblage from layer 3 likely represents a repeatedly visited, seasonal, short-term stone knapping workshop-camp.

The layer 3 assemblage from 2012–2014 excavations (87 m^2^) consists of 409 artefacts that include 38 cores and core-like pieces, 6 CTEs, 277 flakes, 6 blades, 67 shatters, 11 chips, and 4 pebbles. The main part of artefacts is made from silicified limestone, and less is made from dark gray bad-quality flint. Both types of lithic raw materials are found as pebbles, nodules and slabs in several outcrops in the immediate vicinity of the site. The stone knapping technology is characterized by the predominance of Levallois preferential technique, represented by prevailing Levallois preferential cores (19 of 24 identifiable core) that were used for production of large and medium sized flakes. Also, five cores show the presence of Levallois recurrent technique using parallel flaking. Most cores are heavily reduced, and plain and faceted platforms predominate.

Retouched tools include 24 artefacts, with addition of two Levallois flakes and 4 pebbles (defined as hammerstones (3) and retoucher). Retouched notches (31% of total tools), and flakes and fragments with retouch (34%) predominate among retouched tools. Also, fragments of two double side-scrapers made on laminar blanks are represented. Although the layer 3 assemblage in the Darvagchai-Zaliv-1 site shares technological similarity with all early MP industries with Levallois technique defined in the South Caucasus, the very small number of formal retouched tools makes it impossible to provide a reliable attribution of the assemblage to any of these industries.

The Darvagchay-Zaliv-4 open-air site, discovered in 2010, is located also in the Darvagchay River valley, 500 m from Darvagchay-Zaliv-1. The 2011, 2014–2016, and 2017–2019 excavations at Darvagchay-Zaliv-4 (over 65 m^2^) revealed one level (layer 1c) that yielded MP artefacts. The OSL date of 111.9 ± 14.8 ka BP obtained for layer 1c indicates a similar age with MP layer 3 in Darvagchai-Zaliv-1. The MP assemblage from Darvagchay-Zaliv-4 includes only 114 artefacts and is similar to that in Darvagchai-Zaliv-1 [37].

**Tinit-1 open-air site**

The Tinit-1 open-air site in the Rubas River valley, excavated in 2007–2010 (86 m^2^) and 2011 (25 m^2^), represents a frequently visited short-term stone knapping workshop-camp (probably, a short-term hunting camp), like Darvagchai-Zaliv-1, but shows a finely stratified succession of 8 or 11 archaeological horizons within the stratigraphic sequence comprising nine lithological strata [38]. Based on technological and typological features of the lithic assemblage from 2007–2010 excavations (1516 artefacts), the excavators attributed archaeological horizons 1–4 to the Middle/Upper Paleolithic transition and horizons 5–11 to the terminal MP [82].

Several radiocarbon dates on charcoal samples obtained in the University of Arizona AMS Laboratory (USA) for Tinit-1 vary from 39,200 ± 740 BP (AA-93693) for horizon 2 to 47,800 ± 1500 BP (AA-93695) for horizon 10 [38], indicating that all cultural horizons in this site are dated from the late MP, between 42,978 ± 510 calBP (median 42,853 calBP) and 51,131 ± 1917 calBP (median 50,977 calBP; 68.3% probability; OxCal 4.4 Bronk Ramsey 2021, IntCal20 atmospheric curve [83] available at: https://c14.arch.ox.ac.uk/oxcal/OxCal.html), and correlate to MIS 3. Although radiocarbon dates clearly determine the late MP age of the Tinit-1 site, up to the lower limit of radiocarbon dating, the application of other rediometric dating methods may be useful to confirm the radiocarbon age range or result in its revision towards the earlier age.

The archaeological sediments in Tinit-1 comprise isolated charcoal pieces and thin charcoal coatings, as well as several spots containing large (up to 3 cm) charcoal pieces and likely representing disturbed surface hearths were found in horizons 4 and 8 [38]. All artefacts found in Tinit-1 are made from noduls and slabs of flint and silicified limestone, which outcrops are located 1–2 km from the site.

The material from 2011 excavation the excavators divided in two assemblages [38], following the earlier assumption [82] about two different culture-chronological stages represented in this site. Despite both assemblages from 2011 excavation contain small amounts of lithic artefacts (only 80 artefacts in total including 7 retouched tools in the upper archaeological horizons 1–4 and 255 artefacts including 9 retouched tools in the lower horizons 5–9), their technological and typological characteristics are quite similar, indicating that we are dealing with a single MP industry. The 2007–2010 excavations produced over 1600 lithic artefacts that confirm the 2011 excavation results.

For the overall assemblage from 2011 excavation Anoykin et al. [38] note that the stone knapping technology was aimed to the production of elongated blanks using volumetric parallel flaking method and in less degree Levallois recurrent flaking method. All cores have one flaking surface. Refittings of flakes from horizons 2–4, including a most representative refitting consisting of 23 refits (18 flakes and an exhausted core), corroborate these observations. Most flakes (54.4% in horizons 1–4 and 59.3% in horizons 5–9) show the parallel scar pattern on dorsal surfaces, including the bidirectional pattern (15% in horizons 1–4 and 11% in horizons 5–9); the percentage of other dorsal patterns is low. In horizons 1–4, the average blade index (Ilam) is 21% and increases to 29%, if laminar flakes are included. In horizons 5–9, the average Ilam is 17.2%, but increases up to 31% if laminar flakes are included, corresponding to the average values obtained for the upper horizons 1–4. Most flakes (65.2% in horizons 1–4 and 62.2% in horizons 5–9) have plain striking platforms, while faceted and dihedral platforms are less represented (IF is ~17 and IFs = 8.7 in horizons 1–4, and ~15 and 11.2 respectively in horizons 5–9).

The typologically definable retouched tools are few. They comprise single and double side-scrapers, scraper-knives, notches, a fragment of Mousterian point, retouched Levallois point, atypical endscrapers, burin, a truncated-faceted implement, and a few other tools.

**Data 11. Zagros Mousterian in the Lesser Caucasus and Armenian Highlands**

In the South Caucasus, the MP sites known at present are concentrated within two main geographical regions: first, in the south-western Caucasus, which incorporates the Transcaucasian region of Russia, Abkhazia, western Georgia, and South Ossetia, all located in river basins related to the Black Sea marine basin; and second, in the Lesser Caucasus and Armenian Highlands, which represents the southernmost region of the Caucasus that includes mountain areas of western Azerbaijan, the South Georgian volcanic highland in southern Georgia, and the Armenian volcanic highlands in Armenia, all located in the Kura and Araks river basins related to the Caspian Sea marine basin.

All of the Zagros Mousterian sites defined previously in the Caucasus are situated in the Lesser Caucasus and Armenian Highlands. Earlier, various researchers noted similarities between the MP sites in this region and the Zagros Mousterian sites in the Zagros Mountains in Iran [47,84-88], but only in the early 2000s Golovanova and Doronichev [7,8] assigned the MP assemblages from this region to the Zagros Mousterian industry. Golovanova and Doronichev [7] also concluded that the long MP occupational sequences in Taglar cave (Azerbaijan) and Yerevan-1 cave (Armenia) likely show a general succession (called "Yerevan–Taglar tradition") of cultural development of the Lesser Caucasus variant of the Zagros Mousterian industry during the time interval from MIS 4 to the end of Neanderthal occupation of the Caucasus in mid MIS 3, between 70 and 40 ka ago. We also assumed that the first manifestations of Zagros Mousterian in the Caucasus may be dated from the earlier time, presumably from MIS 5a or earlier. Modern studies in the Lesser Caucasus, including new results of radiometric dating in several sites, confirm and supplement these conclusions that we made previously.

Seven stratified MP cave sites that produced Zagros Mousterian assemblages (as summarized in [7,8]) were known in the and Armenian Highlands and Lesser Caucasus until recently. They include Taglar, Zar, Dashsalakhly, and Gazma caves, and layer III in Azykh (Azokh 1) cave in Azerbaijan [85,86,89,90], and Lusakert 1 and Yerevan 1 caves in Armenia [91,92]. Most of them have been discovered and excavated in the 1960s–1980s. Recent excavations were performed only in three of these sites, including Azokh 1 (Azykh) cave [5,93,94], Gazma cave [95], and Lusakert 1 cave [9,96-101]. Two sites, which yielded small assemblages that can be attributed to Zagros Mousterian, have been recently reported in Armenia, in Lusakert 2 cave [97] and Angeghakot-1 rockshelter (84 artifacts; [102])*.* The lithic assemblage from Angeghakot-1 includes 21 retouched points, of which 10 are so called "Yerevan points" (with bases thinned by truncated-faceting method; [100]) that are typical to the Zagros Mousterian industry. A small MP assemblage from Lusakert 2 cave, defined by excavators as a Levallo-Mousterian industry [97], is characterized by Levallois recurrent technique, with a significant amount of Levallois elongated points and blades, and retouched tools that include side-scrapers, end-scrapers, Mousterian points, and a few Yerevan-type points with truncated-faceted bases characteristic of the Zagros Mousterian industry.

Two more sites recently discovered and investigated in Armenia produced large lithic assemblages showing features typical to the Zagros Mousterian industry in this region. The Barozh 12 open-air site, discovered in 2009 and excavated in 2014, yielded a total of 12,549 obsidian artifacts [103]. The lithic assemblage is characterized by the predominance of Levallois recurrent technique, and the tool set that includes besides other tools the retouched Levallois and Mousterian points, and simple and convergent scrapers, among which a retouched point with truncated-faceted base similar to Yerevan points is represented. The **Bagratashen-1 open-air site, discovered in 2009 and preliminary excavated between 2009 and 2011, yielded the MP assemblage comprising a total of 568 lithic artifacts [104]. The lithic assemblage includes Levallois recurrent and blade cores with parallel removals, several Levallois retouched and elongated Mousterian points, and side-scrapers that recall artefact assemblages from other MP sites in the region, and 11 truncated-faceted tools that are typical to the Zagros Mousterian industry in the Lesser Caucasus.**

Nowadays, the MP sequence of Taglar cave, which includes six distinct MP layers, provides one of the most representative data source for characterizing cultural peculiarities, and the dynamics of technological and typological development of the Zagros Mousterian industry in the Lesser Caucasus. The excavator, Djafarov [85,86] defined the MP industry from Taglar cave as a "Taglar-type Mousterian". He characterized this industry as a laminar (Ilam = 25.3–43.7) and faceted (IF = 60.8–73.6; IFs = 22.7–34.4) Typical Mousterian industry of Levallois facies (IL = 38.4–53.1), rich in side-scrapers (in average 54.6% of total tools) and retouched points (in average 39.5% of total tools). Other tools include mainly various notched and denticulated tools, and atypical end-scrapers.

Golovanova and Doronichev [7] attibuted the MP industry from Taglar cave to Zagros Mousterian and noted its similarity to the Zagros Mousterian assemblages from Bisitun and Warvasi caves in Iran in the following features: high percentage of laminar blanks (high Ilam) and prepared platforms on blanks (high IF and IFs), as well as a high percentage of various convergent tools, including Levallois retouched and Mousterian points, elongated convergent and asymmetric angular scrapers (many of which can be defined rather as *déjeté* points or *déjeté* scrapers, or arc-backed knives), and more rare typical *déjeté* scrapers and limaces. The rare narrow and long double scrapers with massive cross-section are also characteristic for the Taglar industry, and represent a characteristic tool type, called "bar-shaped scrapers" or rods, of the Zagros Moustrian industry in Iran. However, the most significant feature of tool-working defined in the Zagros Mousterian industry in Zagros Mountains is the truncated-faceting method. In Taglar, this method was used in the manufacture of convergent scrapers, Mousterian points, and various side-scrapers including so called "scrapers with a thinned body" (defined as the "Taglar-type scrapers"; [85,86]).

The excavator, Yeritsyan [91] designated the MP industry from 7 distinct layers in Yerevan cave as a specific industry type called "Yerevan–type Mousterian", which is characterized by moderate average indexes of Levallois blanks (IL ≤ 20) and faceted platforms (IFs ~ 17–22), and a low blade index (Ilam ~ 6–8) (Table 4), as well as the tool set showing the predominance of side-scrapers and retouched points, and a wide use of truncated–faceting method. In Yerevan cave, this method was applied for thinning bases of retouched points, which Yeritsyan defined as the "Yerevan-type points", side-scrapers including scrapers similar to "Taglar-type scrapers", and some end-scrapers. Later, Djafarov [86] noted the cultural proximity between MP industries from Taglar and Yerevan caves, primarily in the tools manufactured using the truncated–faceting method, but also pointed to significant differences between these industries. He marked the main differences of the Taglar industry, such as the higher indexes of Levallois technique, blades, and prepared and faceted platforms, as well as Levallois tools and side-scrapers, and a much lower percentage of truncated–faceted tools. Golovanova and Doronichev [7] showed that these differences can be explained by the lithic industry development during the time from the Yerevan to Taglar sequence, and identified the assemblage from layer C1 at Lusakert-1 cave as an intermediate stage between the MP sequences in Yerevan and Taglar caves.

Following the earlier proposal by Yeritsyan [91], we dated layer C1 at Lusakert-1 to early MIS 3 [7: fig 2]. The recent results of infrared stimulated luminescence (IRSL) dating indicate that most MP levels in this cave, including the level (or unit) 3 corresponding to former layer C1, are dated from MIS 3, between about 40–60 ka ago [101,105]. Yeritsyan [92] assigned the lithic assemblage from layer CI to a Levalloiso-Mousterian industry and noted its similarity with MP assemblages from the upper MP layers 3 and 4 in Yerevan-1 cave. Golovanova and Doronichev [7: 129, tab. XI] also noted that the layer C1 assemblage has similar high Levallois, blade, faceted platforms, and side-scraper indexes as the lower MP layers at Taglar cave. Retouched points, including Levallois retouched points, and side-scrapers combined comprise about 61% of total retouched tools in layer CI (about 88.4% in Taglar cave), and include both Yerevan-type points with truncated–faceted bases and truncated–faceted scrapers that are characteristic to MP assemblages from both Yerevan and Taglar caves, and the Zagros Mousterian industry in the Lesser Caucasus and Armenian Highlands.

**Data 12. Comparison of MP assemblages from Weasel Cave, Tinit-1 and Saradj-Chuko Grotto with the Zagros Mousterian assemblages in the Lesser Caucasus and Armenian Highlands**

Based on the published data [13-16], we [8,10] defined that the early MP industry from the middle complex (layers 12–21) in Weasel cave is similar to laminar Mousterian industries known in the Southern Caucasus, Levant and Zagros, and especially to the Zagros Mousterian industry in the Lesser Caucasus, which we identified earlier as the Caucasian variant of Zagros Mousterian defined previously in the Zagros Mountains in Iran [7]. The early MP industry from Weasel cave is based on laminar technology, and contains many laminar and Levallois blanks, including rare blades and Levallois points, as well as shows a high number of tools made on laminar flakes and blades. The tool set is characterized by a high percentage of convergent tools that include unretouched and retouched Levallois points, Mousterian points and elongated Mousterian points made on blades, angled (*déjeté*) scrapers, and other convergent tools. Also, rare scraper-knives with retouched backs, narrow and thick bar-like double scrapers (so called “rods”), and truncated-faceted scrapers were found. These technological characteristics and tool types are typical for the Zagros Mousterian assemblages in the Lesser Caucasus. Unfortunately, the absence of published statistical data on MP assemblages from Weasel cave makes it impossible a more detail comparison of these assemblages with the Zagros Mousterian assemblages in the Lesser Caucasus.

The lithic assemblages from all archaeological horizons in Tinit-1 site are dated from late MP (MIS 3, between approximately 43–51 ka calBP), based on radiocarbon dates [38]. However, radiocarbon estimates for the lower archaeological horizons in this site are near the lower limit of radiocarbon dating, suggesting that application of other rediometric dating methods (OSL or TL) may be useful to confirm the radiocarbon age range or revise the lower radiocarbon age limit in this site towards an earlier age.

In Tinit-1, Anoykin et al. [38] defined the application of the volumetric parallel flaking method and in less degree the Levallois recurrent flaking method. The average indexes of blades (Ilam ~17–21, but increases to ~29–31, if laminar flakes are included), and prepared and faceted platforms (IF ~15–17 and IFs ~9–11) are not high and fluctuate quite strongly in different horizons. This may be partly explained by a specialized character of the site, in which short-term stone knapping workshops and hunting camps are defined in all archaeological horizons. Apparently, the best blanks and functional tools could have been taken from Tinit-1 to somewhere.

The Zagros Mousterian assemblages dating from late MP (MIS 3) in the Lesser Caucasus show the higher indexes of blades, and prepared and faceted platforms. The typologically definable tools are few in Tinit-1, and comprise single and double side-scrapers, scraper-knives, atypical endscrapers, and rare Levallois, retouched Levallois and Mousterian points, angled scrapers, and truncated-faceted tools. All these tool types are typical for Zagros Mousterian.

The new material from recent excavations in Saradj-Chuko grotto in the Elbrus region, in the western part of the north-central Caucasus, confirms our previous estimation of the early MP industry from Weasel cave [8,10], and supplements the data on the features and spread of the Zagros Mousterian industry in the North Caucasus. The assemblages from layers 6B and 6A at Saradj-Chuko grotto show the presence of laminar technology based on flaking of laminar flakes and blades from non-Levallois cores.

The blade index (Ilam) is low in both layers (10.7 in layer 6B and 5.2 in layer 6A), but increases to 27.2 and 45.1 respectively, if laminar flakes are included. The indexes of prepared and faceted platforms are high in layers 6B and 6A (IF = 42.7 and 45.5, and IFs = 37 and 38.8 respectively). Basing on these technological indicators, the early MP industry from layers 6B and 6A at Saradj-Chuko grotto can be characterized as a laminar, faceted Mousterian.

Like the later MP industry from Tinit-1, an indicative feature of the earlier MP industry from layers 6B and 6A in Saradj-Chuko grotto is the abundance of laminar or bladey flakes, with length between 1.5 and 2 times their width [22], and herewith the rarity of both true blades and typical triangular Levallois flakes or points. Unlike the MP industry from Tinit-1, high indexes of prepared and faceted platforms is the feature of the MP industry from Saradj-Chuco grotto, having analogy in the Zagros Mousterian industry in the Lesser Caucasus.

The Zagros Moustrian industry in the Lesser Caucasus is characterized not only by the developed laminar flaking technology, but also the predominance of narrow blades among elongated blanks or blades (with L ≥ 2 m). In layer 6B at Saradj-Chuko grotto, very narrow blades (5–10 mm in width; or bladelets) and narrow blades (10–15 mm in width) predominate, while wider blades are rare. The thickness of bladelets/blades from layer 6B varies from 1 to 5 mm, but most are 1–3 mm thick. Also, very elongated blanks, with length between 2.5 and 4 times their width, are typical for the Zagros Moustrian industry in the Lesser Caucasus. For example, such blanks compose 85.7% of total elongated blanks in Taglar cave. In layer 6B at Saradj-Chuko grotto such blanks compose 60.8% of total elongated blanks. In other MP industries in the South Caucasus, very elongated blanks typically compose less than half of total elongated blanks.

A typological comparison of the layer 6B assemblage from Saradj-Chuko grotto with the Zagros Mousterian assemblages indicates that in all of these assemblages the unifacially worked tools absolutely predominate, and the main categories of retouched tools are various convergent tools, including Levallois retouched and Mousterian points, convergent scrapers and scraper-knives, and *déjeté* points and *déjeté* scapers, and rare limaces, as well as simple side-scrapers [18,19]. There are also rare diagonal and transverse scrapers, and various endscrapers and burins. Tools with ventral thinning retouch are typical, and truncated-faceted pieces are especially indicative.

These comparative data indicate that the MP assemblages from all well-stratified sites known at present in the eastern half of the North Caucasus, including Weasel cave, Tinit-1 and Saradj-Chuko grotto, share common technological and typological features that show the highest similarity with the Zagros Moustrian assemblages in the Lesser Caucasus, which, in turn, show a high degree of similarity with the Zagros Moustrian industry in the Zagros Mountains, in Iran. The same time, the laminar character of the stone knapping technology and the peculiarities of the tool set composition distinguish these MP sites of the Eastern Caucasus from the Eastern Micoquian industry, which is well represented in the north-western Caucasus [10]. The technological and typological features of the MP assemblages in the north-central and north-eastern Caucasus indicate a cultural connection of these assemblages with the Zagros Mousterian industry in the Lesser Caucasus and Zagros Mountains. This evidence suggests that the Mousterian industry of the Eastern Caucasus represents a variant of the Zagros Mousterian industry.

**Data 13. Archaeological evidence of contacts between the Eastern Micoquian and Zagros Mousterian populations of Neandertals in the North Caucasus**

**Mezmaiskaya Cave**

In Mezmaiskaya cave, three obsidian artefacts were found in layers 3 (two retouched flakes) and 2B4 (chip) ([39]; Fig 2: 14,15). The XRF analysis of two obsidian artefacts from layer 3 indicated that both artefacts originate from the Zayukovo (Baksan) obsidian source in the Elbrus region, located ~200–250 km south-east from Mezmaiskaya [39,72]. In layer 3, both obsidian retouched flakes were found in excavation horizon 2 on square R-21, where 12 artefacts were found in total, including 6 flakes and the two retouched flakes made from obsidian. The microstratigraphic, planigraphic and faunal analyses indicate that during the time of layer 3 Neanderthals repeatedly occupied and intensively inhabited Mezmaiskaya cave for some time. Also, microstratigraphic and planigraphic data show that the area of obsidian artefacts discovery is not related with any of the most intensively inhabited by Neanderthals parts of the cave interior.

**Saradj-Chuko grotto**

The evidence in support of contacts between the Neanderthal populations in the Elbrus region and in the north-western Caucasus is also found in Saradj-Chuko grotto. In layer 6B in Saradj-Chuko grotto, there were found five bifacial tools (Fig 9) that are not characteristic to Zagros Moustrian, but are typical to the Eastern Micoquian assemblages in the north-western Caucasus. The microstratigraphic and planigraphic analyses indicate that the main concentration of lithic artefacts in layer 6B is related to the lower part of the layer, which is about 20 cm thick, and coincides with the distribution of bones on most of the excavation area. The artefacts and bones show especially dense concentration on quadrants R–S – 9–12 inside the grotto, where they are associated with two surface hearths. The microstratigraphic analysis also indicates that the main concentration level in layer 6B likely represents a palimpsest of at least two occupation episodes that were separated by a short time interval.

All the tools characteristic to the Eastern Micoquian industry of the north-western Caucasus were found within the main concentration of lithic artefacts in layer 6B (Fig 9). The tools include two wide triangular small handaxes shaped using a plano-convex method; a small bifacial leaf point with a double-convex cross-section shaped using alternative retouch (dorsal retouch along one side, and dorsal and then ventral retouch along another side); and two asymmetrical bifacial scrapers (or scraper-knives) shaped using a plano-convex method, one of which with an unretouched back. Three of the tools are made from Zayukovo obsidian, suggesting that they were produced locally (within or in close proximity to the Zayukovo obsidian source area), but two tools are made from a beige flint and a silicified limestone, which sources are not identified [19]. A traceological analysis of two of the tools, a bifacial leaf point and one of the asymmetrical bifacial scraper-knives, indicates that the first tool is a projectile tip that bears traces of high-velocity impact resulted from its use as a projectile weapon and also traces of its supplementary or secondary use as a stone retoucher; and the second tool was used as a meat knife. All five bifacial tools found in layer 6B are pointed instruments that could potentially served as hunting mobile inventory, which hunters used for hunting animals or butchering hunting prey.

The low occurrence of typical Eastern Micoquian tools in layer 6B at Saradj-Chuko grotto implies that the making of these bifacial tools was not an important part of the tool-making behavior in this area, but also that Eastern Micoquian Neanderthal groups did not advance this far east. However, the finding of these tools in a Zagros Mousterian site suggests that small, mobile hunting groups of Eastern Micoquian Neanderthals would enter the Zagros Mousterian cultural area, but only sporadically made this, moving as far east as the Saradj-Chuko grotto located at the western boundary of this cultural area. The data from Saradj-Chuko grotto confirms the assumption, made earlier basing on the soursing of obsidian artefacts from Mezmaiskaya [39], about sporadic contacts between the two culture-different Neanderthal populations, Eastern Micoquian Neanderthals in the western North Caucasus and Zagros Moustrian Neanderthals in the eastern North Caucasus, that simultaneously occupied the North Caucasus during the MP period, from late MIS 5 to mid MIS 3.

**Supporting Information References**

1. Lioubine VP. La grotte mousterienne Barakaevskaia (Nord Caucase). L’Anthropologie. 1998; 102(1): 67–90.
2. Liubin VP. Paleolit Kavkaza (Paleolithic of Caucasus). In Paleolit Kavkaza i Severnoi Azii, Serya Paleolit mira, Leningrad; 1989, pp. 8–142. **(In Russian)**
3. Beliaeva EV. Mustierskiy mir Gubskogo ushelia (Severnii Kavkaz)*(* The Mousterian World of the Gubs Gorge), St.Petersburg; 1999. **(In Russian)**
4. Ščelinskij VE. Der Mittelpalaolithische fundplatz Ilskaya II im Westlichen Kubangebiet*.* In: Jahrbuch des Romisch-Germanischen Zentralmuseums Mainz. 45; (1998).
5. Golovanova LV, Levkovskaya GM, Baryshnikov GF. Le Nouveau Site Mousterien en Grotte de Matouzka, Caucase Septentrional (Resultats des Fouilles de 1985–1987). L’Anthropologie. 1990; 94: 739–762.
6. Golovanova L, Hoffecker J, Nesmeyanov S, Levkovskaya G, Kharitonov V, Romanova G, et al. Site du Micoque Esteuropeen du Caucase de Nord (Resultats presentifs des etudes de la grotte Mezmaiskaya, les fouilles des annes 1987-1993). L’Anthropologie. 1998; 1: 45–66.
7. Golovanova L, Hoffecker J, Kharitonov V, Romanova G. Mezmaiskaya Cave: A Neanderthal Occupation in the Northern Caucasus. Current Anthropology. 1999; 40(1): 77–86.
8. Ponce de Leon MS, Golovanova L, Doronichev V, Romanova G, Akazawa T, Kondo O, et al. Neanderthal brain size at birth provides insights into the evolution of human life history. PNAS. 2008; 105(37): 13764–13768.
9. Petr M, Hajdinjak M, Fu Q, Essel E, Rougier H, Crevecoeur I, et al. The Evolutionary History of Neandertal and Denisovan Y Chromosomes. Science. 2020; 369(6511): 1653–1656.
10. García-Martínez D, Bastir M, Gómez-Olivencia A, Maureille B, Golovanova L, Doronichev V, et al. Early development of the Neanderthal ribcage reveals a different body shape at birth compared to modern humans. Science Advances. 2020; 6(41): eabb4377.
11. Chevalier T, Colard T, Colombo A, Golovanova L, Doronichev V, Hublin J-J. Humeral trabecular bone ontogeny in Neandertals and recent modern humans. J. of Human Evol. 2021; 154: 102968
12. Filippov AK, Liubin VP. Bone retouchers from the Mousterian layer and spatial distribution of cultural remains. Neanderthals of the Gups Gorge in the North Caucasus. Maykop, 1994. (In Russian)
13. Nesmeyanov SA. Geomorphological aspects of the mountain Paleolithic paleoecology (on the example of the Western Caucasus). Moscow, 1999. (In Russian)
14. Baryshnikov G, Hoffecker J, Burgess R*.* Palaeontology and Zooarchaeology of Mezmaiskaya Cave (Northwestern Caucasus, Russia). J. of Archaeol. Science. 1996; 23(3): 313–335.
15. Golovanova LV, Doronichev VB, Kulkova MA, Cleghorn N, Sapelko TV. Significance of ecological factors in the Middle to Upper Paleolithic transition. Current Anthropology. 2010; 51(5): 655–691.
16. Lebedev VA, Vashakidze GT. Quaternary volcanoes of the Greater Caucasus and their cataloging based on geochronological, volcanic and isotope-geochronological data. Volcanology and seismology. 2014; 2: 29–45.
17. Behrensmeyer AK. Taphonomic and ecologic information from bone weathering. Paleobiology. 1978; 4: 150–162.
18. Hoffecker JF, Cleghorn N. Mousterian Hunting Patterns in the Northern Caucasus and the Ecology of the Neanderthals. Intern. J. of Osteoarch. 2000; 10: 368–378.
19. Cleghorn N. A zooarchaeological perspective on the Middle to Upper Paleolithic transition at Mezmaiskaya Cave, the Northwestern Caucasus, Russia. Unpublished PhD thesis. 2006. Stony Brook University, New York.
20. Golovanova LV, Doronichev VB, Levkovskaya GM, Lozovoy SP, Nesmeyanov SA, Pospelova GA, et al. Matuzka Cave. 2006. St. Petersburg, Ostrovityanin. **(In Russian)**
21. Rasmussen KL, De La Fuente G, Bond A, Mathiesen K, Vera S. Pottery firing temperatures:a new method for de termining the firing temperature of ceramics and burnt clay. J. of Archaeol. Science. 2012; 39, 1705–1716.
22. Jordanova N, Jordanova D, Mokreva A, Ishlyamski D, Georgieva B. Temporal changes in magnetic signal of burnt soils–a compelling three years pilot study. Sci. Total Environ. 2019; 669, 729–738.
23. Doronicheva EV. Mobilnost naseleniya I ekspluatatsiya kamennogo sir’ya na stoyankah vostochnogo mikoka Severnogo Kavkaza (The mobility of human groups and raw material exploitation at Eastern Micoquian sites in the Northern Caucasus). In: Ragimova VN, Djafarov AG, Zeinalov AA (Eds.), Karabah v kamennom veke. Materiali mejdunarodnoi nauchnoi konferentsii, posvyashennoi 50-letiyu otkritiya paleoliticheskoi peshernoi stoyanki Azyh v Azerbaidjane (3-7 oktyabrya 2010 g., Baku, Azerbaidjanskaya Respublika). 2010. Baku, Teknur, pp. 129–147. **(In Russian)**
24. Doronicheva EV. Siryeviye strategii drevnego cheloveka v srednem paleolite na Severo-Zapadnom Kavkaze. (Raw material strategies in the Northwestern Caucasus Middle Paleolithic). Vestnik of the St-Petersburg State Univer. 2011; 2(3), 192–199. **(In Russian)**
25. Doronicheva EV, Kulkova MA. Petrograficheskoye issledovaniye kremnya iz mestorojdeniy I stoyanok srednego paleolita na Severo-Zapadnom Kavkaze. (Petrography study of flint from the natural outcrops and Middle Paleolithic sites in the Northwestern Caucasus). Stratum Plus. 2011; 1, 153–169. **(In Russian)**
26. Doronicheva E, Kulkova M, Grégoire S. La grotte Mézmayskaya (Caucase de Nord): exemple de l’utilisation des matières premières lithiques au Paléolithique Moyen et Supérieur. L’Anthropologie. 2012; 116(3), 378–404.
27. Doronicheva EV, Shackley MS. Obsidian exploitation strategies in the Middle and Upper Paleolithic of the Northern Caucasus: New data from Mezmaiskaya cave. PaleoAnthropology. 2014; 2014, 565–585.
28. Doronichev VB. Analysis of stone knapping technology in the Early Palaeolithic: the problem of method. Soviet Archaeology. 1991; 3, 130-142. **(In Russian)**
29. Doronichev VB, Golovanova LV. Non-Levallois bifacial assemblages with Levallois cores – an eastern “anomaly” into a nice picture. J. of the Intern. Union for Prehistoric and Protohistoric Sci. 2021; 3-2, pp. 38–62.
30. Boëda E. Le concept Levallois: Variabilité des méthodes. 1994. Paris, CNRS Éditions.
31. Boëda E. Levallois: A volumetric construction, methods, a technique. In: Dibble HL, Bar-Yosef O (Eds.), The definition and interpretation of Levallois technology, Monographs in World Archaeology 23. 1995. Madison, WI, Prehistory Press, pp. 41–68.
32. Derevianko AP, Anoikin AA, Zenin VN, Leshchinsky SV. Ranniy paleolit Yugo-Vostochnogo Dagestana. 2009. Novosibirsk: Izd. IAET SO RAN. **(In Russian)**
33. Liubin VP, Belyaeva EV. Middle Paleolithic sites of Ingushetia and the problem of Paleolithic migrations in the central part of the Greater Caucasus. Stratum plus. 2001; 1, 322–337. **(In Russian)**
34. Kotovich VG. Kamennyi vek Dagestana. 1964. Makhachkala, Dag. filial Inst. istorii, yazyka i literatury AN SSSR. **(In Russian)**
35. Anoikin АА. Coastal Dagestan Middle Paleolithic Marine Izotope Stage 5 Assemblages: Regional Context. Vestnik NSU. Series: History and Philology. 2018; 17(7), 74–85. **(In Russian)**
36. Rybalko AG, Kandyba AV. Issledovaniya srednego paleolita Zapadnogo Prikaspiya (po materialam pamyatnika Darvagchay-zaliv-1). Gumanitarnye nauki v Sibiri. 2019; 26(2): 5–10. **(In Russian)**
37. Derevianko AP, Amirkhanov KA, Zenin VN, Anoikin AA, Rybalko AG. Problemy paleolita Dagestana. 2012. Novosibirsk, Izd. IAET SO RAN. **(In Russian)**
38. Reimer P, Austin W, Bard E, Bayliss A, Blackwell P, Ramsey B, et al. The IntCal20 Northern Hemisphere radiocarbon age calibration curve (0–55 cal kBP). Radiocarbon. 2020; 62.
39. Liubin VP. Ranniy paleolit Kavkaza (Early Paleolithic of the Caucasus). In: Paleolit SSSR, Serya Arkheologiay SSSR. 1984. Moscow, pp. 45–93. **(In Russian)**
40. **Djafarov AK. Mustierskaya kultura Azerbaidjana (po materialam Taglarskoi pesheri) (The Mousterian Culture of Azerbaidjan (on materials of Taglar cave). 1983. Baku.** **(In Russian)**
41. Djafarov AG. Middle Paleolithic of Azerbaijan. 1999. Baku. **(In Russian)**
42. Kozlowski JK. The Middle and the early Upper Paleolithic around the Black Sea. In: Akazawa et al. (Eds.), Neanderthals and Modern Humans in Western Asia. 1998. Plenum Press, New York, pp. 461–482.
43. Beliaeva EV, Lioubine VP. The Caucasus—Levant—Zagros: Possible relations in the Middle Paleolithic. In: Otte M (Ed.), Anatolian Prehistory at the Crossroads of Two Worlds. 1998. Vol. I, ERAUL 85, Liege, pp. 39–55.
44. Guseinov MM. The Palaeolithic in Azerbaijan (Early Palaeolithic). In: Kamenniy vek i eneolit v Azerbaijane. 1984. Baku. (In Russian)
45. Guseinov MM. The Early Palaeolithic in Azerbaijan. 2010. Baku. (In Russian)
46. Yeritsyan BG. Yerevan cave site and its place among the earliest sites of Caucasus. Abstract of Ph.D. Dissertation. 1970. Moscow. (In Russian)
47. Yeritsyan BG. A new Lower Paleolithic cave site of Lusakert I (Armenia). Kratkie soobsheniya Instituta Archeologii. 1975; 141: 54-67 (In Russian).
48. Asryan L, Olle A, Moloney N, King T. Lithic assemblages of Azokh Cave (Nagorno Karabagh, Lesser Caucasus): raw materials, technology and regional context. J. of Lithic Studies*.* 2014; 1(1). doi: 10.2218/jls.v1i1.775
49. Fernández-Jalvo Y, King T, Andrews P, Yepiskoposyan L, Moloney N, Murray J, et al. The Azokh Cave complex: Middle Pleistocene to Holocene human occupation in the Caucasus. J. of Human Evol*.* 2010; 58: 103–109.
50. Djafarov AG, Zeynalov AA, Avsharova IN. Archaeological excavation in Gazma Palaeolitic cave. Archaeological research in Azerbaydjan*.* 2010: 26–30 (In Russian)
51. Adler DS, Yeritsyan B, Wilkinson K, Pinhasi R, Bar-Oz G, Nahapetyan S, et al. The Hrazdan gorge Palaeolithic project, 2008-2009. In: Avetisyan P, Bobokhyan A (Eds.), Archaeology of Armenia in Regional Context. 2012. Yerevan, pp. 21-37.
52. Fourloubey C, Beauval D, Colonge D, Liagre J, Ollivier V, Chataigner C. Le paléolithique en Arménie: état de connaissances acquises et données récentes. Paléorient*.* 2003; 29: 5–18.
53. Frahm E, Schmidt BA, Gasparyan B, Yeritsyan B, Karapetian S, Meliksetian K, et al. Ten seconds in the field: rapid Armenian obsidian sourcing with portable XRF to inform excavations and surveys. J. of Archaeol. Science. 2014; 41: 333–348.
54. Frahm E, Feinberg JM, Schmidt-Magee BA, Wilkinson KN, Gasparyan B, Yeritsyan B, et al. Middle Palaeolithic toolstone procurement behaviors at Lusakert Cave 1, Hrazdan valley, Armenia. J. of Human Evol. 2016; 91: 73–92.
55. Gasparyan B, Egeland CP, Adler DS, Pinhasi R, Glauberman P, Haydosyan H. The Middle Paleolithic Occupation of Armenia: Summarizing Old and New Data. In: Gasparyan B, Arimura M (Eds.), Stone Age of Armenia. A Guide-book to the Stone Age Archaeology in the Republic of Armenia*.* 2014. Kanazawa University Press, pp. 65–105.
56. Srivastava A. Single-aliquot and Single-grain Infrared Stimulated Luminescence (IRSL) Dating of sediments at Lusakert, Armenia. Unpublished PhD thesis. 2015.
57. Liagre J, Gasparyan B, Ollivier V, Nahapetyan S. Angeghakot I (Armenia) and the Identification of the Mousterian Cultural Facies of “Erevan points” Type in the Southern Caucasus. Paleorient. 2006; (32.1).
58. Glauberman P, Gasparyan B, Wilkinson K, Frahm E, Raczynski-Henk Y, Haydosyan H, et al. Introducing Barozh 12: A Middle Palaeolithic Open-Air Site on the Edge of the Ararat Depression, Armenia. Aramazd. Armenian journal of Near Eastern studies. 2015; IX(2): 7–20.
59. **Egeland CP, Gasparian B, Fadem CM, Nahapetyan S, Arakelyan D, Nicholson CM. Bagratashen 1, a stratified open-air Middle Paleolithic site in the Debed river valley of northeastern Armenia: a preliminary report. Archaeol. Research in Asia. 2016; 8: 1–20.**
60. **Brittingham A, Hren MT, Hartman G, Wilkinson KN, Mallol C, Gasparyan B, et al.** Geochemical Evidence for the Control of Fire by Middle Palaeolithic Hominins. Scien. Reports. 2019: 9: 15368*.*
